# A revision of the *Phelisterhaemorrhous* species group (Coleoptera, Histeridae, Exosternini)

**DOI:** 10.3897/zookeys.854.35133

**Published:** 2019-06-10

**Authors:** Michael S. Caterino, Alexey K. Tishechkin

**Affiliations:** 1 Department of Plant & Environmental Sciences, Clemson University, Clemson, SC 29634 USA Clemson University Clemson United States of America; 2 California Dept. of Food and Agriculture Plant Pest Diagnostics Center, Sacramento, CA 95832 USA California Dept. of Food and Agriculture Plant Pest Diagnostics Center Sacramento United States of America

**Keywords:** Histerinae, Neotropical biodiversity

## Abstract

The *Phelisterhaemorrhous* species group is established here, revising the seventeen included species, four of which are described as new. This group is named for and contains the type species of *Phelister*, so represents a core around which a modern concept of the dumping-ground genus *Phelister* may be developed. The group includes several common and well-known species in the Americas, including some of the only *Phelister* to exhibit distinctive coloration. Several of these are typically found in cattle dung, and have likely expanded beyond their native ranges as cattle spread throughout the Americas. The group contains the following species: *Phelisterhaemorrhous* Marseul, 1854, *Phelisteraffinis* J.E. LeConte, 1859, *Phelisterparallelisternus* Schmidt, 1893, *Phelistermobilensis* Casey, 1916, *Phelisterbrevistriatus* Casey, 1916, *Phelistersonorae***sp. nov.**, *Phelisterwarneri***sp. nov.**, *Phelisterpuncticollis* Hinton, 1935, *Phelistersubrotundus* (Say, 1825), *Phelisterrouzeti* (Fairmaire, 1850), *Phelisterrufinotus* Marseul, 1861, *Phelisterthiemei* Schmidt, 1889, *Phelisterparecis***sp. nov.**, *Phelisterbryanti***sp. nov.**, *Phelistervernus* (Say, 1825), *Phelisterchilicola* Marseul, 1870, and *Phelisterbruchi* Bickhardt, 1920. We also designate the following new synonymies: *Phelisterhaemorrhous* Marseul (= *Phelisterrubicundus* Marseul, 1889, **syn. nov.**); *Phelistersubrotundus* (= *Phelistercontractus* Casey, 1916, **syn nov.**); *Phelisterrouzeti* (Fairmaire) (= *Phelisterfairmairei* Marseul, 1861; **syn. nov.**, = *Phelisterwickhami* Casey, 1916, **syn. nov.**); *Phelisterrufinotus* Marseul, 1861 (= *Epierusmarseulii* Kirsch, 1873, **syn. nov.**); and *Phelisterthiemei* Schmidt, 1889 (= *Phelisterstercoricola* Bickhardt, 1909, **syn. nov.**).

## Introduction

With 91 described species, and many more undescribed, the largely Neotropical genus *Phelister* Marseul is one of the most species rich genera in the family Histeridae. Since its description it has served as a taxonomic dumping ground for a great diversity of small, generally non-descript Exosternini, even including some from outside the Neotropical realm, and phylogenetic analyses have revealed it to be para- and polyphyletic (Caterino and Tishechkin 2015). Recent revisions of some other Neotropical exosternine genera have marginally improved *Phelister*’s coherence by removing a number of species (to *Operclipygus* Marseul and *Baconia* Lewis (Caterino and Tishechkin 2013a, 2013b, respectively). However, exactly how to define *Phelister* itself has not become much clearer, and substantial additional phylogenetic work is needed to more fully understand relationships among the species currently included. Relationships to other as-yet-unrevised genera such as *Pseudister* Bickhardt, *Nunbergia* Mazur, and *Conchita* Mazur remain particularly obscure.

Beyond the phylogenetic questions surrounding *Phelister*, identification of species in the group is practically impossible without reference to type specimens. No comprehensive (or even partial) keys exist aside from some very local (e.g., Casey 1916) or outdated (Bickhardt 1916) treatments, and the species are very difficult to distinguish based solely on external characters in any case. Yet, accurate identification of some of the species of *Phelister* has practical significance, as some species have been cited in forensic investigations (e.g., *P.rufinotus* Marseul; Aballay et al. 2013) and others have been studied with regard for their potential to control of dung-breeding flies (*P.panamensis* LeConte; Summerlin et al. 1991), *P.rufinotus* and *P.haemorrhous* Marseul (Koller et al. 2002). The species (presumably all predatory) exhibit an interesting array of ecological associations, ranging from loose synanthropy to mammalian inquiliny to myrmecophily. Many also occur in great abundance and may make up substantial fractions of pitfall and flight intercept trap collections in the neotropics. So improved identification efficiency would significantly benefit biodiversity inventory work.

To begin to address these systematic impediments, we plan to revise the species of *Phelister* over a series of smaller treatments of putatively closely related groups of species. Here we begin with a group of 17 species loosely centered on the now firmly-established type of the genus, *Phelisterhaemorrhous* Marseul. Previous confusion over the type species of *Phelister* was resolved by the ICZN following an application to recognize Kryzhanovskij and Reichardt’s (1976) designation of *P.haemorrhous* as the type (Caterino and Tishechkin 2013c, ICZN 2015). Our hypothesis of monophyly for this group is based on only a few shared characters (discussed more below), and the group does contain considerable diversity. But together they seem to represent a near continuum of forms, with many of them quite difficult to distinguish. The group is largely, though not completely supported as monophyletic in our recent global analysis of *Phelister* (Caterino and Tishechkin 2015). We attribute the exceptions mostly to some inadequacies of search algorithms (across over 750 taxa), as well as a lack of molecular data for the majority of relevant species. Possible relationships among the species are discussed in further detail below, based on some more thorough analyses of a subset of taxa.

The species we attribute to the informal *P.haemorrhous* group include some of the most commonly encountered *Phelister* species in both North and South America. Several are associated with the dung of domestic cattle and horses. Additionally, the group includes nearly all of the *Phelister* species currently known to occur in the Nearctic realm. Interestingly, although the group’s species collectively span the Americas, they are sparse in the wet tropics, and the species are most often encountered in the northern and southern subtropical/temperate zones. Furthermore, several of the species exhibit some red coloration, which has drawn an unusual amount of attention to them. Given these attributes, it is a relatively well-described group, with only a few new species, all of which exhibit fairly limited distributions and, in several cases, narrow ecological associations.

## Materials and methods

### Specimens

Type material of all species was examined by one or both of the authors. Other specimens examined were assembled from a large number of institutions:

**AKTC** Alexey Tishechkin Collection, Sacramento, USA

**ASUC** Arizona State University Collection, Tempe, USA

**NHMUK** Natural History Museum, London, UK

**CASC** California Academy of Sciences Collection, San Francisco, USA

**CDFA** California State Collection of Arthropods, Sacramento, USA

**CEMT** Coleção de Entomologia, Universidade Federal do Mato Grosso, Cuiabá, Brazil

**CHJG** Jeffrey P. Gruber Collection, Madison, USA

**CHND** Nicolas Degallier Collection, Paris

**CHPWK** Peter Kovarik Collection, Columbus, USA

**CHSM** Slawomir Mazur Collection, Warsaw, Poland

**CMNC** Canadian Museum of Nature, Ottawa, Canada

**CMNH** Carnegie Museum of Natural History, Pittsburg, USA

**CUAC** Clemson University Arthropod Collection, Clemson, USA

**DVC** David Verity Collection, Long Beach, CA

**DZUP** Departamento de Zoologia, Universidade Federal do Paraná, Curitiba, Brazil

**FMNH** Field Museum, Chicago, USA

**FSCA** Florida State Collection of Arthropods, Gainesville, USA

**INBIO** Instituto Nacional de Biodiversidad, San Jose, Costa Rica

**LSAM** Louisiana State Arthropod Museum, Baton Rouge, USA

**MCZC** Museum of Comparative Zoology, Harvard University, Cambridge, USA

**MNHN** Museum National d’Histoire Naturelle, Paris, France

**MSCC** Michael Caterino Collection, Clemson, USA

**MSNG** Museo Genova di Storia Naturale “Giacomo Doria”, Genova, Italy

**MTD** Staatliches Museum für Tierkunde, Dresden, Germany

**SEMC** Snow Entomology Museum, University of Kansas, Lawrence, USA

**TAMU** Texas A&M University Collection, College Station, USA

**UNESP** Universdade Estadual Paulista, Faculdade de Engenharia de Ilha Solteira, Ilha Solteira, Brazil

**USFQ** Universidad San Francisco de Quito, Ecuador

**USNM** National Museum of Natural History, Washington, USA

**WSUC** Washington State University Insect Collection, Pullman, USA

**ZMHB** Zoological Museum of Humboldt University, Berlin, Germany

We present brief diagnostic descriptions for most species, focusing on those character systems in which differences among species are typically found. They are not intended to be exhaustive descriptions of each species’ morphology. We have attempted to make most of them consistent in character content and order, facilitating comparison as well as their reuse of descriptions in other contexts. The ‘remarks’ sections highlight the few most important key characters of each species. Much of the morphological terminology used is based on Wenzel and Dybas (Wenzel and Dybas 1941), but modified to follow more recent treatments (Helava et al. 1985, Ohara 1994, Kanaar 1997, Lawrence et al. 2011). We have presented an extensive discussion of Exosternini-specific morphological terminology in Caterino and Tishechkin (2013a), and refer the reader to the labeled illustrations there.

Material examined lists provide verbatim data only for holotypes and lectotypes, and summary data for all other material of described species, whether paratypes or non-type localities. Most of these represent lists of states or provinces within countries. For US states, counties are included. Within verbatim records, data are enclosed in double quotes, with data on separate labels separated by a slash ‘/’.

Conventional imaging was done using a Visionary Digital’s ‘Passport’ portable imaging system, which incorporates a Canon D7 with MP-E 65mm 1–5×macro zoom lens. Images were stacked using Helicon Focus software (HeliconSoft, Kharkiv, Ukraine). SEM imaging was done on a Zeiss EVO 40 scope. Most specimens were sputter coated with gold, though some uncoated specimens were examined in ‘variable pressure’ mode. Following histerid conventions, total body length is measured from the anterior margin of the pronotum to the posterior margin of the elytra (to exclude preservation variability in head and pygidial extension), while width is taken at the widest point, generally near the elytral humeri. Ten specimens were measured wherever possible.

### Phylogenetic analyses

We reanalyzed a subset of taxa from the 750+ taxon data set of Caterino and Tishechkin (2015) to attempt to better resolve species within *Phelister* and to evaluate the level of support for the *haemorrhous* group as we delimit it here. This pruned data set included only small numbers of exemplars for those groups previously strongly supported as monophyletic. Specifically, it includes only four species of *Baconia*, two species of *Hypobletus*, two species of *Operclipygus*, and single exemplars of some other smaller but previously supported genera outside *Phelister*. We also reduced the number of outgroups to six (from 61). This reduced data set included a total of 231 taxa, including all described and undescribed *Phelister* and *Pseudister* spp., as well as many other new species of uncertain placement. All taxa were scored for 260 morphological characters. Approximately one-fourth were represented by some molecular data, including some combination of 18S (937 characters for 62 spp.), 28S (993 characters for 32 spp.), and cytochrome oxidase I (679 characters for 63 spp). We did not realign the length variable portions for this reduced dataset, maintaining homology assessments from the preceding analysis. For original alignment parameters see Caterino and Tishechkin (2015). This reduced data set is available as an online supplement (Suppl. material 1). Tree searching was performed in PAUP* (v. 4.0a164; Swofford 2002) under the maximum parsimony criterion, running 1000 random sequence addition replicates, saving no more than 2500 trees for each replicate.

## Taxonomy

### The *Phelisterhaemorrhous* group

**Diagnosis.** Recognizing members of the *P.haemorrhous* group is difficult, given the general similarity prevailing throughout *Phelister*. However, most members exhibit most or all of the following character states. There is as yet no single and simple (non-homoplasious) synapomorphy to which we can point, even in genitalic morphology:

• Both mandibles have a strong tooth. A tooth on the right mandible is common outside the *P.haemorrhous* group; having both teeth is relatively uncommon;

• Outer subhumeral elytral stria present, but rarely in more than apical half. Many otherwise similar species have the outer subhumeral longer;

• Elytral striae 1–4 complete, 5^th^ stria variable (but when abbreviated usually represented by a basal puncture); sutural stria abbreviated;

• Elytra often with rufescent maculae. There are very few bicolored species of *Phelister* outside this group;

• Body form elongate, less rounded than many *Phelister*;

• Lateral portion of pronotal disk bearing coarser punctures. More rarely present outside this group;

• Labrum broad, often weakly emarginate;

• Postmesocoxal stria usually well developed, ending freely or recurved anteriad to mesepimeron;

• 1^st^ abdominal ventrite with complete inner and abbreviated outer postmetacoxal stria;

• Males of several species have the pronotal keel more densely punctate than the females; most of these species are red-maculate;

• Aedeagus usually simple, with apices often variably separated; rarely with ventral dentate process;

• Median lobe with proximal apodemes divided into fine proximal and thick distal portions;

• Male eighth tergite lacking basal accessory sclerites;

• Most species occurring in temperate to seasonal subtropical areas, with few species known from wet tropics.

### Checklist of the species

***Phelisterhaemorrhous* Marseul, 1854**

*Phelisteregenus* Marseul, 1854b

*Phelisterrubicundus* Marseul, 1889c, **syn. nov.**

***Phelisteraffinis* JE LeConte, 1859**

*Phelistersimplex* Casey, 1916

*Phelistersolator* Marseul, 1861

***Phelisterparallelisternus* Schmidt, 1893**

***Phelistermobilensis* Casey, 1916**

***Phelisterbrevistriatus* Casey, 1916**

***Phelistersonorae*, sp. nov.**

***Phelisterwarneri*, sp. nov.**

***Phelisterpuncticollis* Hinton, 1935**

***Phelistersubrotundus* (Say, 1825)**

*Phelisterrubricatus* Lewis, 1908

*Phelistersayi* Carnochan, 1915b

*Phelisterfrosti* Carnochan, 1915b

*Phelistercarnochani* Casey, 1916

*Phelistercontractus* Casey, 1916, syn. nov.

***Phelisterrouzeti* (Fairmaire, 1850)**

*Phelisterfairmairei* Marseul, 1861, syn. nov.

*Phelisterwickhami* Casey, 1916, syn. nov.

*Phelisterpimalis* Casey, 1916

*Phelisteraztecanus* Casey, 1916

***Phelisterrufinotus* Marseul, 1861**

*Epierusmarseulii* Kirsch, 1873, syn. nov.

***Phelisterthiemei* Schmidt, 1889**

*Phelisterstercoricola* Bickhardt, 1909, syn. nov.

***Phelisterparecis*, sp. nov.**

***Phelisterbryanti*, sp. nov.**

***Phelistervernus* (Say, 1825)**

*Phelistersaunieri* Marseul, 1861

***Phelisterchilicola* Marseul, 1870**

***Phelisterbruchi* Bickhardt, 1920**

### Key to Species

**Table:** 

1	Protarsal claws modified, strongly bent at base, then straight (at least in males; Fig. 8C)	**2**
–	Protarsal claws simple	**5**
2	Fifth elytral stria complete	**3**
–	Fifth elytral stria abbreviated	**4**
3	Lateral pronotal stria absent; male prosternal carinal striae separate anteriorly, nearly or fully reaching anterior margin; metaventrite lacking distinct patches of punctures anteriad metacoxae; only known from Argentina	*** P. bryanti ***
–	Lateral pronotal stria present; male prosternal carinal striae meeting short of anterior margin, delimiting a small space (Fig. 5B); metaventrite with distinct patches of punctures anteriad metacoxae; northeastern Brazil	*** P. puncticollis ***
4	Mesometaventral stria extending anteriad to midline of mesoventrite (Fig. 4E); frontal stria complete; only known from Sonora, Mexico	*** P. sonorae ***
–	Mesometaventral stria barely extending anteriad mesometaventral suture; frontal stria interrupted across much of middle of frons; elytral striae thin and finely impressed; elytra frequently with diffuse rufescent patches (Fig. 9B); known only from Chile	*** P. chilicola ***
5	Lateral pronotal stria more or less complete, extending well posteriad pronotal midpoint	**6**
–	Lateral pronotal stria abbreviated (not extending posteriad pronotal midpoint) or absent	**8**
6	Frontal stria complete	*** P. parecis ***
–	Frontal stria interrupted, usually broadly	**7**
7	Fifth elytral stria usually complete; body slightly larger and rounder (Fig. 6A); aedeagus expanded, rounded apically (Fig. 2I); Nearctic	*** P. subrotundus ***
–	Fifth elytral stria usually abbreviated; body smaller and more elongate (Fig. 6E); aedeagus narrower, almost straight in profile (Fig. 2J); mostly in Mexico, Central America, and northern South America, just extending into the southwestern US	*** P. rouzeti ***
8	Lateral pronotal stria present but abbreviated, restricted to anterior half of pronotal margin; elytra (but not pygidia) often with red markings; southern neotropics and south temperate areas; aedeagus expanded apically, nearly spoon-shaped (Fig. 2K)	*** P. rufinotus ***
–	Lateral pronotal stria absent, rarely detectable as disconnected punctures around anterior angle; red markings, if present, extending to pygidia	**9**
9	Elytra with reddish markings	**10**
–	Elytra unicolorous, usually black, rarely rufescent	**11**
10	Reddish markings extending onto pygidia and venter of apical abdominal segments (Fig. 1A); postmesocoxal stria recurved anteriad to mesepimeron (Fig. 1C); frontal stria frequently broadly interrupted across frons (Fig. 1B); larger, body length 1.85–2.3mm; widespread in Americas	*** P. haemorrhous ***
–	Reddish markings (if present) restricted to elytra; postmesocoxal stria shorter, ending freely; frontal stria more nearly complete; smaller, body length 1.30–1.77mm; restricted to subtropical South America	*** P. thiemei ***
11	Posterior ends of prosternal keel striae parallel and united before base (Fig. 3B); 5^th^ dorsal elytral stria complete; posterior ends of inner metaventral striae tending to recurve mediad in front of metacoxae; south-central US into Mexico	*** P. parallelisternus ***
–	Posterior ends of prosternal keel striae ending freely; 5^th^ dorsal elytral striae only very rarely complete; distribution varied	**12**
12	Epistoma with lateral marginal striae connecting to frontal stria at sides (Fig. 3C); mesometaventral stria distinctly more crenulated than marginal mesoventral stria (Fig. 3E); south-central and southeastern US	*** P. mobilensis ***
–	Epistoma without lateral marginal striae; mesometaventral stria various	**13**
13	Mesometaventral stria absent from middle (Fig. 9D); sutural and 5^th^ dorsal striae absent; restricted to subtropical South America	*** P. bruchi ***
–	Mesometaventral stria complete across middle; sutural and 5^th^ dorsal striae present; North America	**14**
14	Frontal stria nearly complete, often interrupted, or obscured by punctures at middle; southwestern US	**15**
–	Frontal stria more broadly interrupted at middle or interrupted at middle and at sides	**16**
15	Fifth elytral stria well impressed in at least apical half; body piceous; dorsal and ventral striae normally impressed; free-living, southern Arizona to Central America	*** P. brevistriatus ***
–	Fifth dorsal stria weak to absent; entire body rufescent; elytral and ventral striae more finely impressed; portions of ventral striae often effaced; middle and hind tibiae slender and only weakly spinose (Fig. 4G); likely a mammal burrow inquiline; only known from Arizona to Texas	*** P. warneri ***
16	Frontal stria interrupted over antennal bases and at middle, represented at front by distinct, isolated lateral fragments (Fig. 1E); vestiges of lateral pronotal stria often detectable around anterior pronotal corners; ground punctation of metaventrite relatively fine and sparse (contrast elsewhere not as distinct); postmesocoxal stria longer, directed more posterolaterad; south-central US to Central America	*** P. affinis ***
–	Frontal stria usually absent across middle, though fine lateral fragments may be present; lateral pronotal stria not represented by anterior vestiges; ground punctation of pronotum, frons, and metaventrite unusually conspicuous (Fig. 9A); postmesocoxal stria short and turned out behind coxa; widespread in Nearctic	*** P. vernus ***

### Species treatments

#### Phelister haemorrhous

Taxon classificationAnimaliaColeopteraHisteridae

Marseul, 1854

Figs 1 , 2 ; Map 1

Phelister haemorrhous Marseul, 1854: 476.
Phelister egenus Marseul, 1854: 480; Mazur 1984.
Phelister rubicundus Marseul, 1889: cxlvi; syn. nov.

##### Type material.

**Lectotype**, hereby designated: “Phelister haemorrhous M., Italie?, Digot” [the question mark is written on the label]/ “Museum Paris, Coll. de Marseul 2842-90”/ “Type”/ “Lectotype Phelister haemorrhous Marseul, 1853, M.S.Caterino and A.K.Tishechkin des. 2010”, MNHN.

Types of synonyms. *Phelisterrubicundus* Marseul, 1889: **Lectotype**, hereby designated: “Am. Mer” / “Phelister rubicundus Mars Type” / “Lectotype Phelister rubicundus Marseul, 1889, M.S.Caterino and A.K.Tishechkin des. 2010”, NHMUK. *Phelisteregenus* Marseul, 1854b: **Lectotype**, hereby designated: “Carthagena”, MNHN; Paralectotype with same data in NHMUK.

##### Diagnostic description.

Length: 1.85–2.29 mm (avg. 2.03 mm); width: 1.58–1.89 mm (avg. 1.69 mm). Body elongate-oval, widest behind humeri, humeri slightly wider than base of pronotum; anterior of body black, posterolateral corners of elytra, pygidia, legs, and terminal abdominal ventrites distinctly reddish; entire dorsum finely punctulate, the pronotum more densely so than the elytra; frons finely punctulate, impressed along midline, supraorbital stria complete, frontal stria interrupted at middle, slightly sinuate at sides; labrum wide, distinctly emarginate apically; both mandibles with strong tooth on inner edges; pronotum lacking lateral and anterior submarginal striae; pronotal disk with larger punctures interspersed with finer punctures along lateral thirds; elytron with single, complete epipleural stria, outer subhumeral stria present in apical third, inner subhumeral stria absent, dorsal striae 1–4 complete, stria 5 complete or abbreviated at base, and sutural stria obsolete in basal third, diverging from the suture anteriad; propygidium with distinct secondary punctures separated by slightly greater than their widths, denser at sides; pygidium more finely punctate; prosternal keel with two complete striae, finely united by anterior arch, free, diverging posteriorly, finely punctulate between in both sexes; mesoventral marginal stria complete, weakly crenulate, continued at sides by postmesocoxal stria which recurves more or less evenly to anterolateral corner of metaventrite; mesometaventral stria complete, crenulate at middle, curving posteriad to near inner corner of metacoxa; first abdominal ventrite with single, complete lateral stria; protibia with apex obliquely truncate, outer margin weakly rounded, bearing ca. six evenly spaced marginal spines; meso- and metatibiae weakly expanded to apex, mesotibia with ca. five marginal spines, more prominent toward apex, metatibia with distinct spines confined to apical fourth. Aedeagus with basal piece ca. one-fourth total length; tegmen widest just beyond middle, narrowed to apex, apices thin, with deep apical emargination; median lobe short, simple.

**Figure 1.**
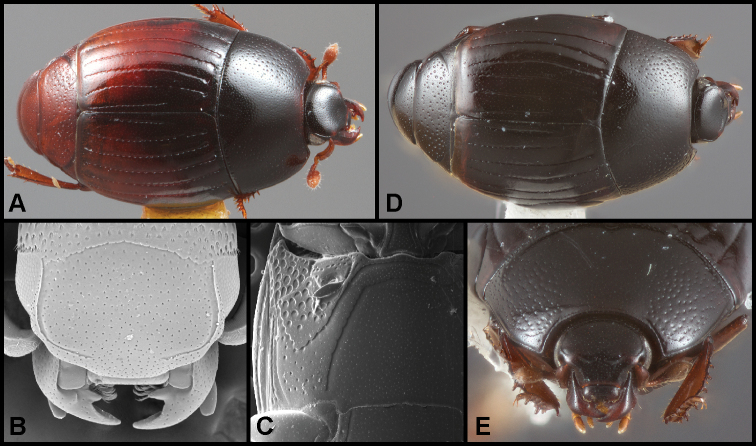
**A–C***Phelisterhaemorrhous* Marseul: **A** Dorsal habitus **B** Head showing frontal and supraorbital striae and mandibular teeth **C** Meso- and metaventrites showing complete, recurved postmesocoxal stria **D–E***Phelisteraffinis* JE LeConte **D** Dorsal habitus **E** Anterior view of head and pronotum showing fragmented frontal stria and rudiments of sublateral pronotal stria.

**Figure 2.**
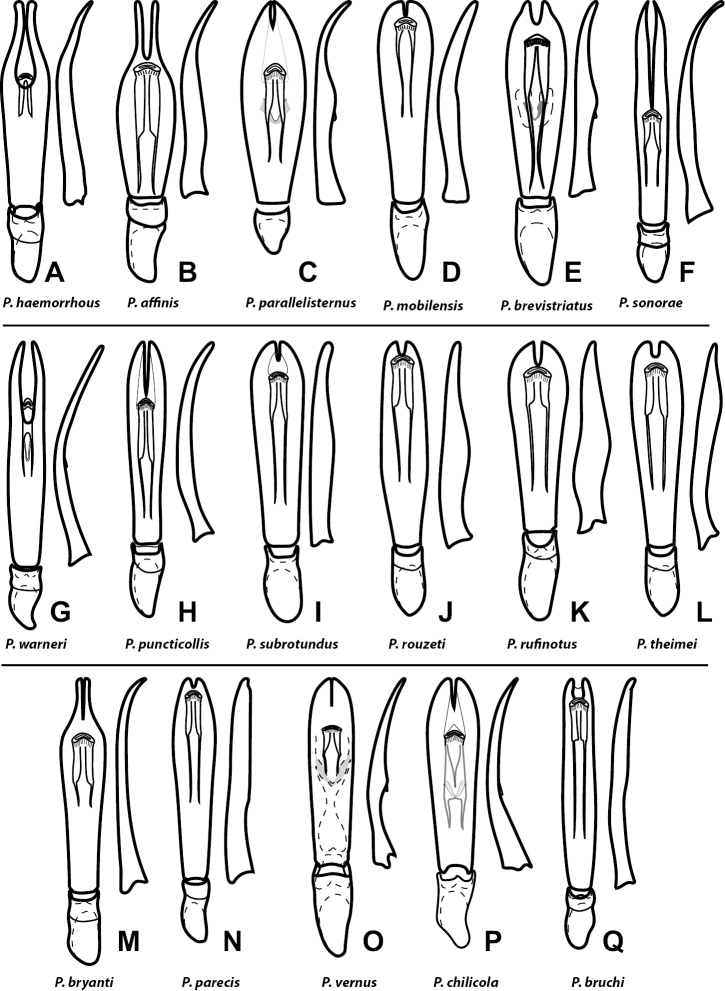
Male genitalia of all *P.haemorrhous* group species, dorsal and lateral views: **A***Phelisterhaemorrhous* Marseul **B***Phelisteraffinis* JE LeConte **C***Phelisterparallelisternus* Schmidt **D***Phelistermobilensis* Casey **E***Phelisterbrevistriatus* Casey **F***Phelistersonorae* sp. nov. **G***Phelisterwarneri* sp. nov. **H***Phelisterpuncticollis* Hinton **I***Phelistersubrotundus* (Say) **J***Phelisterrouzeti* (Fairmaire) **K***Phelisterrufinotus* Marseul **L***Phelisterthiemei* Schmidt **M***Phelisterbryanti* sp. nov. **N***Phelisterparecis* sp. nov. **O***Phelistervernus* (Say) **P***Phelisterchilicola***Q***Phelisterbruchi* Bickhardt.

##### Remarks.

As the type of the genus *Phelister*, *P.haemorrhous* is both typical in many respects, and somewhat unusual. Its distinctive posterior elytral and abdominal coloration is unmistakable, and this, in combination with dentate mandibles, a complete, recurved postmesocoxal stria, complete lateral pronotal stria, and presence of conspicuous lateral pronotal punctures, will easily distinguish it. There are cases where the reddish color is obscure, but the other characters in combination should still allow it to be recognized.

While the species’ type locality is in Europe (Italy), it is clearly a Neotropical species. There is some uncertainty whether it was ever, in fact, collected in Europe. Vienna (1980) and Penati (2009) specifically dismiss its alleged (e.g., Mazur 1984, 1997) occurrence in Sardinia, and Penati (2009) furthermore makes a strong case that specimens reported from Sardinia as *P.haemorrhous*, in fact, represent a species of *Epierus* (Tribalinae). Earlier, Auzat (1925) suggested that no previous records of *Phelister* for Europe were *bona fide* (neither *P.haemorrhous* nor *P.rouzeti*). The label on the type of *P.haemorrhous* does include a question mark after ‘Italie’, and it seems most likely that Marseul received this in a mixed shipment and never, himself, believed the specimen to have originated in Italy.

This species was recently designated to be the type of *Phelister* following the suppression of an inadvertent designation of a *Baconia* species as *Phelister*’s type (Caterino and Tishechkin 2013c; ICZN 2015).

##### Biology.

This species is most commonly encountered in cattle dung. It has also been collected in pitfall traps using a few other types of bait, including human and pig dung. A few specimens have been taken in more general situations, in rotting vegetation (compost) and under the bark of rotten trees.

##### Distribution.

*Phelisterhaemorrhous* is among the more widespread *Phelister* species, extending from Argentina into the southern United States, and possibly having been introduced into Europe (see caveats above). This wide distribution almost certainly owes to its common association with cattle dung, and it has probably expanded its range with cattle production in the New World. **Records: ARGENTINA**: Buenos Aires, Chaco, Córdoba, Corrientes, Entre Rios, Formosa, La Pampa, La Rioja, Mendoza, Misiones, Salta, San Juan; **BELIZE**: Orange Walk; **BOLIVIA**: Bení, Santa Cruz; **BRAZIL**: Amazonas, Ceará, Goias, Maranhão, Mato Grosso, Mato Grosso do Sul, Minas Gerais, Pará, Paraná, Rio Grande do Sul, Rondônia, Santa Catarina, São Paulo, Tocantins; **COLOMBIA**: Amazonas, Antioquia, Cauca, Cundinamarca, Magdalena, Tolima, Valle de Cauca; **COSTA RICA**: Guanacaste, Puntarenas; **CUBA**: Habana, Sierra Bonilla; **DOMINICAN REPUBLIC**: Monte Cristi, Samaná; **ECUADOR**: Guayas, Napo; **EL SALVADOR**: San Salvador; **FRENCH GUIANA**: Cayenne, Sinnamary, St. Laurent du Maroni; **GRENADA; GUATEMALA**: Bobas; **HAITI**: Pic la Selle, Trouin; **JAMAICA**: Kingston; **MEXICO**: Chiapas, Veracruz; **NICARAGUA**: Granada, Region Norte Autónomo; **PANAMA**: Colón, Panamá; **PARAGUAY**: Alta Paraguay; **PERU**: Ucayali; **SAINT VINCENT & GRENADINES**: Saint Vincent; **SURINAME**: Pará, Saramacca; **TRINIDAD & TOBAGO**: St. George, Trinidad; **URUGUAY**: Canelones, Colonia, Montevideo, Salto, San José; **VENEZUELA**: Aragua, Capital, Sucre; **USA: Alabama**: Mobile; **Arizona**: Maricopa; **Florida**: Alachua, Franklin; **Georgia**: Tifton, Wheeler; **Louisiana**: East Baton Rouge, Iberville; **Mississippi**: Harrison, Jackson, Oktibbeha; **South Carolina**: Beaufort, Spartanburg; **Texas**: Brazos, Burleson, Cameron, Gillespie, Hidalgo, Nueces, Wood.

**Map 1.**
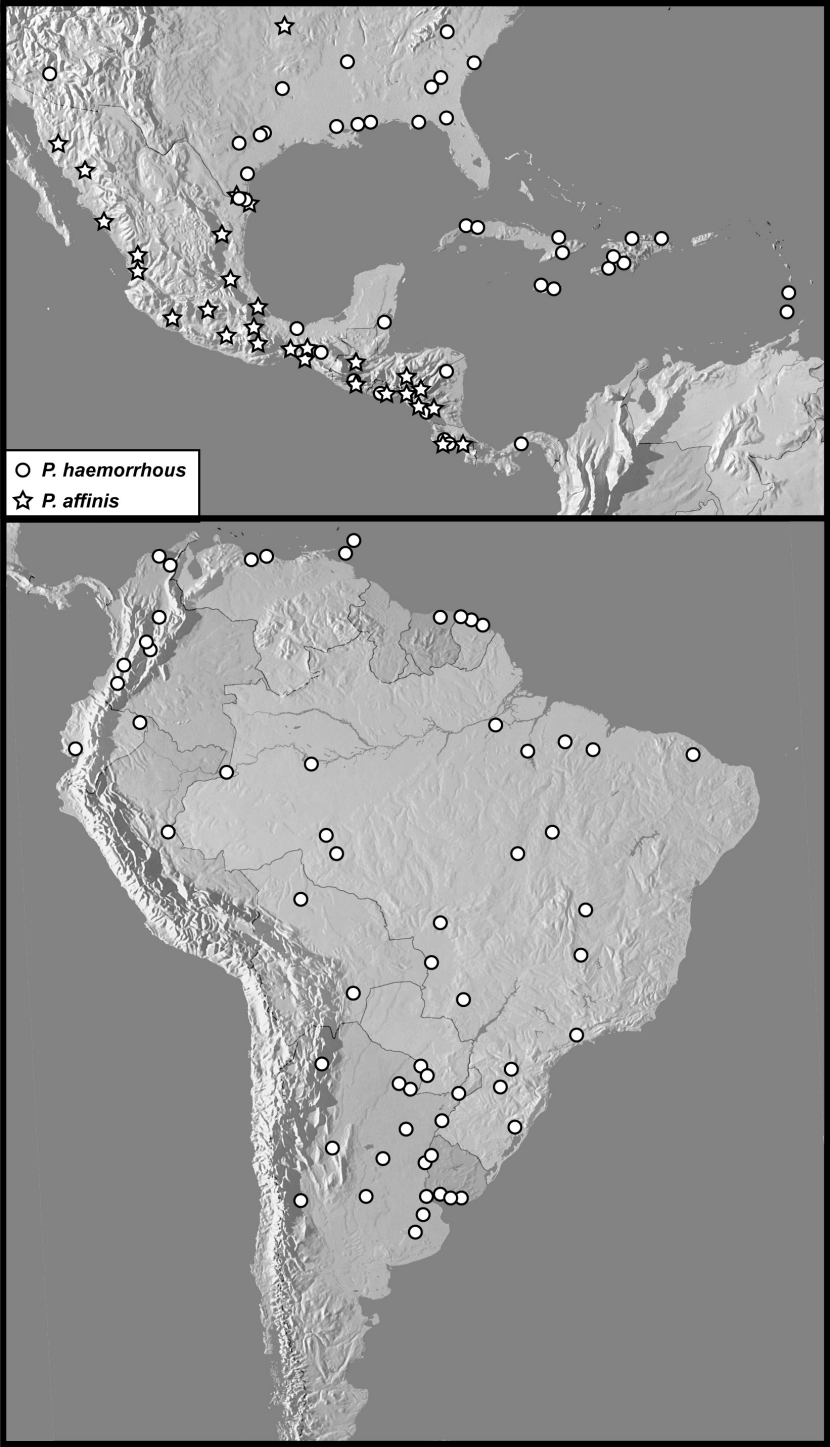
Collecting records for *Phelisterhaemorrhous* (circles) and *P.affinis* (stars).

#### Phelister affinis

Taxon classificationAnimaliaColeopteraHisteridae

JE LeConte, 1859

Figs 1 , 3 ; Map 1

Phelister affinis JE LeConte, 1859: 311.
Phelister simplex Casey, 1916: 230; Mazur 1997.
Phelister solator Marseul, 1861: 164; Marseul 1870.

##### Type material.

Neotype male, hereby designated: “Tejeria, Veracruz, MEX, VII:4:41” / “Col. by H. Dybas” / “Collection R. L. Wenzel” / “Phelister #68 det R.Wenzel” / “Compared with type Phelister affinis LeC. RLW’51; see type notes under solator”; dissected by Rupert Wenzel (FMNH). The type(s) of this species aren’t known, despite searches in the most likely repository (MCZC) and others (FMNH, CMNH, USNM), and despite the apparent fact that Wenzel studied a supposed type in 1951 (labels on specimen). Due to the extreme similarity among members of *Phelister*, we feel that a Neotype designation is necessary to anchor a specific concept for this species.

Types of synonyms. **Lectotype** of *Phelistersolator* Marseul, of undetermined sex, hereby designated: “Phelister solator, Mexic. Sallé 20” / “Coll. Desbordes” / “TYPE”, MNHN. **Lectotype** of *Phelistersimplex* Casey, of undetermined sex, hereby designated: “Lee Co Tex” / “Casey bequest 1925” / “TYPE USNM 38453” / “simplex Csy”, USNM.

##### Diagnostic description.

Length: 1.73–2.01 mm (avg. 1.94 mm); width: 1.50–1.73 mm (avg. 1.65 mm). Body elongate-oval, widest behind humeri, humeri slightly wider than base of pronotum; body more or less uniformly piceous; entire dorsum finely punctulate, the pronotum more densely so than the elytra; frons finely punctulate, impressed along midline, supraorbital stria complete, frontal stria interrupted at sides and at middle, slightly sinuate laterally; labrum wide, distinctly emarginate apically; both mandibles with strong tooth on inner edges; pronotum usually with distinct fragments of submarginal stria in anterior corners; pronotal disk with larger punctures interspersed with finer punctures along lateral thirds; elytron with single, complete epipleural stria, outer subhumeral stria present in apical third, inner subhumeral stria absent, dorsal striae 1–4 complete, stria 5 present in apical half-two-thirds, very rarely complete, but nearly always with a basal puncture, and sutural stria obsolete in basal third, diverging from the suture anteriad; propygidium with distinct secondary punctures separated by slightly greater than their widths; pygidium more finely punctate; prosternal keel with two complete striae, finely united by anterior arch, free, diverging posteriorly, finely punctulate between in both sexes; mesoventral marginal stria complete, smooth, continued at sides by postmesocoxal stria which runs posteriad two-thirds of the distance to metepipleuron; mesometaventral stria complete, weakly crenulate to smooth, angulate mediad mesocoxa, extending posteriad to near inner corner of metacoxa; first abdominal ventrite with single, complete lateral stria; protibia with apex obliquely truncate, outer margin weakly rounded, bearing ca. six evenly spaced marginal spines; meso- and metatibiae weakly expanded to apex, mesotibia with ca. five marginal spines, more prominent toward apex, metatibia with distinct spines confined to apical fourth. Aedeagus with basal piece a little over one-fourth total length; tegmen widest just beyond middle, abruptly narrowed to thin, divided apices; median lobe more than half tegmen length, proximal apodemes thin near base, thickened toward gonopore.

**Figure 3.**
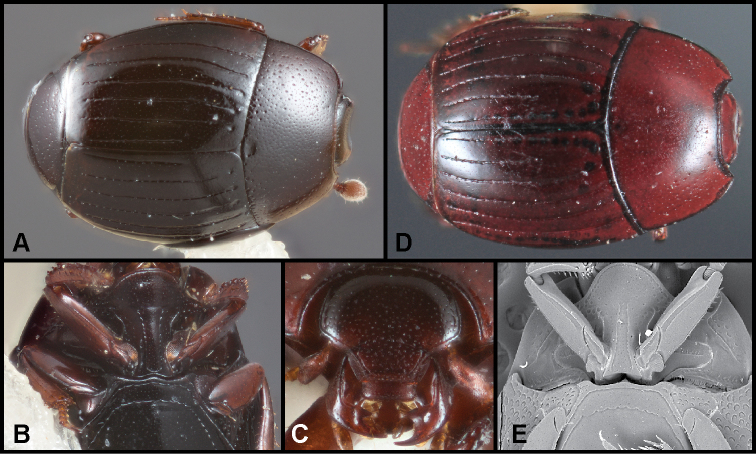
**A–C***Phelisterparallelisternus* Schmidt: **A** Dorsal habitus **B** Ventral view showing prosternal and meso- and metaventral striae **C–E***Phelistermobilensis* Casey: **C** Frontal view showing complete epistomal stria **D** Dorsal habitus **E** Ventral view (SEM) showing prosternal and meso- and metaventral striae.

##### Remarks.

This species was previously synonymized with *Phelistercontractus* Casey by Mazur (1997), in error. Having studied its type, we instead synonymize *P.contractus* with *P.subrotundus* Say (below).

##### Biology.

Label data indicate rather generalist habitat preferences, having been collected in cow, horse, and gopher tortoise dung, under decayed leaves, in rotting breadfruit, in fire-scorched *Yucca* L., and in rotten *Opuntia* Miller, and the species even exhibits some facultative myrmecophily, with records from nests of both *Acromyrmex* Mayr and *Azteca* Forel ants.

##### Distribution.

This species occurs from Central America through Mexico, just into the south-central United States. **Records: COSTA RICA**: Guanacaste, Puntarenas, San José; **EL SALVADOR**: La Libertad, San Salvador; **GUATEMALA**: Baja Verapaz, Escuintla, Santa Rosa; **HONDURAS**: Choluteca, Francisco Morazán; **MEXICO**: Chiapas, Colima, Hidalgo, Michoacán, Morelos, Nayarit, Oaxaca, Sinaloa, Sonora, Tamaulipas, Veracruz; **NICARAGUA**: Granada, León, Madriz, Managua, Zelaya; **USA: Oklahoma**: Latimer; **Texas**: Cameron, Hidalgo.

#### Phelister parallelisternus

Taxon classificationAnimaliaColeopteraHisteridae

Schmidt, 1893

Figs 2 , 3 ; Map 2

Phelister parallelisternus Schmidt, 1893b: 86.

##### Type material.

**Lectotype** of undetermined sex, hereby designated: “Mexico” / “parallelisternus Sch.” / “parallelisternum [sic]” / “Type” / “coll. J.Schmidt” / “LECTOTYPE Phelister parallelisternus Schmidt 1893, M.S. Caterino & A.K. Tischechkin des. 2010” (ZHMB); one paralectotype of undetermined sex is also designated at ZMHB: “Mexico”, and one at MNHN: “Mexique, col. Bickhardt”.

##### Diagnostic description.

Length: 1.65–1.93 mm (avg. 1.78 mm); width: 1.22–1.58 mm (avg. 1.46 mm). Body elongate-oval, widest at humeri, humeri slightly wider than base of pronotum; body uniformly dark rufescent to piceous; frons finely but distinctly punctulate, somewhat depressed along midline; supraorbital stria complete, frontal stria interrupted across middle; labrum wide, distinctly emarginate apically; both mandibles with strong tooth on inner edges; pronotum with coarse ground punctation throughout, with larger punctures becoming more densely intermingled toward sides; prescutellar impression distinct; lateral and anterior marginal pronotal striae continuous, complete, slightly crenulate at front; submarginal pronotal striae absent; elytron with single, complete, crenulate epipleural stria, outer subhumeral stria present in apical one-third to one-half, inner subhumeral stria absent, dorsal striae 1–5 complete (5^th^ rarely abbreviated from base); sutural stria present in posterior three-fourths; ground punctation of pygidium and propygidium similarly fine, propygidium with secondary punctures small, sparse, mostly separated by two to three times their widths; prosternal lobe with truncate to weakly emarginate anterior margin, with fine marginal stria; prosternal keel with two complete striae parallel and distinctly united by basal arch, variably connected anteriorly, with faint, secondary basal striae nearer procoxa, finely punctulate between in both sexes; mesoventrite distinctly projecting, marginal stria complete, crenulate, continued at sides by postmesocoxal stria which runs posteriad nearly to metepipleuron; mesometaventral stria complete, weakly crenulate, arched weakly onto basal one-fourth of mesoventrite, extended posteriad by lateral metaventral stria to near inner corner of metacoxa; metaventral disk with few secondary punctures anteromediad metacoxae; first abdominal ventrite with complete inner lateral stria, outer lateral stria nearly complete, often fragmented at base; protibia with apex obliquely truncate, outer margin weakly rounded, bearing 5–6 marginal spines; meso- and metatibiae weakly expanded to apex, mesotibia with ca. five marginal spines, more prominent toward apex, metatibia with distinct spines confined to apical fourth. Aedeagus with distinct ventral process, just basad middle, basal piece short, less than one-fourth total length; tegmen with sides rounded, widest just beyond middle, apices narrowly separated, apical emargination narrow and shallow; median lobe approximately one-half tegmen length, proximal apodemes thin near base, thickened toward gonopore.

##### Remarks.

This species is quite similar to *P.affinis*, but can be consistently distinguished by its parallel and rather narrowly separated prosternal striae, and its complete 5^th^ dorsal stria. Its frontal stria is interrupted at the middle, but not at the sides, and it never appears to show any indication of a sublateral pronotal stria.

##### Biology.

The only indications of habits for this species come from a few Oklahoma specimens sifted from ‘bay’ litter. A couple of specimens were also collected using flight interception traps.

##### Distribution.

The known distribution of this species is oddly disjunct. The types and other early specimens (NHMUK) bear no more specific locality than Mexico. Yet all recent specimens we have seen are from the south-central United States, specifically Oklahoma, Arkansas, and Missouri. It is most surprising that no specimens have been seen from Texas. **Records: USA: Arkansas**: Faulkner; **Missouri**: Pike; **Oklahoma**: Latimer.

**Map 2.**
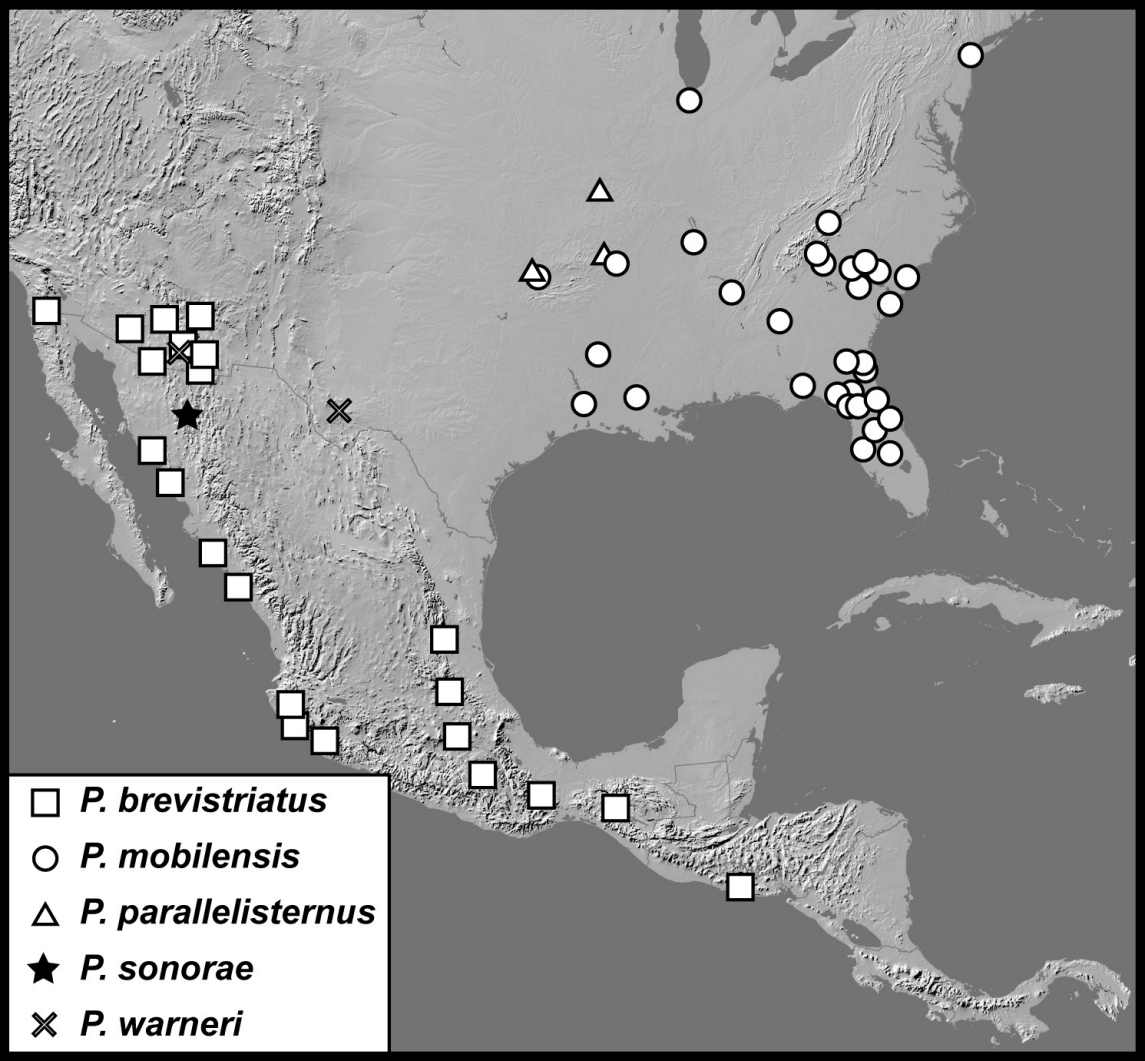
Collecting records for *Phelisterbrevistriatus* (squares), *P.mobilensis* (circles), *P.parallelisternus* (triangles), *P.sonorae* (star), and *P.warneri* (diagonal cross).

#### Phelister mobilensis

Taxon classificationAnimaliaColeopteraHisteridae

Casey, 1916

Figs 2 , 3 ; Map 2

Phelister mobilensis Casey, 1916: 232.

##### Type material.

**Lectotype**, hereby designated: “Ala. Mobile” / “Casey bequest 1925” / “Type USNM 38450” / “mobilensis Csy.” / “Lectotype Phelister mobilensis Casey, M.S.Caterino and A.K.Tishechkin des. 2010”, USNM. Four paralectotypes: two with same data as type, two from “Pensacola, Fla.”, all USNM.

##### Description.

Length: 1.85–2.05 mm (avg. 1.93 mm); width: 1.58–1.73 mm (avg. 1.59 mm). Body uniformly dark rufescent to piceous, elongate oval, widest just behind midline; frons finely but distinctly punctulate; supraorbital stria complete, fine across vertex; frontal stria well impressed along inner margin of eyes, fragmentary to obsolete along upper epistomal margin, but continued anteriad along lateral and, generally, apical margins of epistoma; labrum transverse, at most weakly emarginate apically; mandibles both with strong tooth along incisor margin; pronotum strongly narrowed anteriorly, very finely punctate at middle but rather coarsely so in lateral thirds; prescutellar impression distinct; lateral and anterior marginal striae continuous, the anterior diverging slightly from the margin and crenulate; lateral submarginal striae absent; elytra with single, complete epipleural striae; outer subhumeral stria present in apical half only; inner subhumeral stria absent; dorsal elytral striae 1–4 complete, 5^th^ present in apical half only, sutural stria just slightly longer than 5^th^; propygidium almost uniformly coarsely punctate, punctures separated by less than their widths; pygidium much more finely and sparsely punctate; prosternal lobe shape evenly rounded, with marginal stria obsolete toward sides; prosternal keel narrowed from base to apex; keel striae separate basally, converging between coxae, narrowly separated, parallel anteriad; anterior mesoventral margin weakly produced; marginal mesoventral stria complete, weakly crenulate; mesometaventral stria arching weakly onto basal third of mesoventrite; metaventral disk weakly punctate, with lateral stria nearly complete to inner corner of metacoxa; postmesocoxal stria extending posterolaterad, mostly straight, ending short of apex of metepisternum; abdominal ventrite 1 with one complete and one fragmentary lateral striae, impunctate at middle, increasingly punctate to sides; protibia with apex truncate, outer margin weakly rounded, bearing ca. five prominent, evenly spaced marginal spines; meso- and metatibiae weakly expanded to apex, mesotibia with ca. five marginal spines, more prominent toward apex, metatibia with distinct spines along most of margin; aedeagus gradually widened toward apex, apically rounded with short, narrow apical emargination; median lobe with simple basal apodemes, ca. half tegmen length.

##### Remarks.

*Phelistermobilensis* exhibits one highly distinct character, a stria on the epistoma lining its anterior and lateral margins, which is continuous with the lateral portion of the otherwise interrupted frontal stria. Other unusual characters include particularly spinose front and middle tibiae, and a meso-metaventral stria which is distinctly more crenulate than the marginal mesoventral stria.

##### Biology.

Label data indicate quite generalized habitat preferences, with records from dung (dog and chicken), decaying vegetation, *Geomys* Rafinesque burrows, and fungi. Specimens have also been collected using flight interception traps and Lindgren funnel traps.

##### Distribution.

This species is found primarily near the Gulf and Atlantic coasts of the southeastern United States. Records from Indiana and New York could use additional confirmation. **Records: USA: Alabama**: Russell, Shelby; **Arkansas**: Pulaski; **Florida**: Alachua, Duval, Gilchrist, Highlands, Hillsborough, Lake, Leon, Levy, Marion, Nassau, Orange, Polk, Putnam; **Georgia**: Burke, Charlton; **Indiana**: Lake; **Louisiana**: Calcasieu; East Baton Rouge, Natchitoches; **New York**: [state record only - AMNH]; **North Carolina**: Transylvania; **Oklahoma**: Latimer; **South Carolina**: Aiken, Anderson, Bamberg, Barnwell, Beaufort, Georgetown, Oconee, Richland; **Tennessee**: Benton.

#### Phelister brevistriatus

Taxon classificationAnimaliaColeopteraHisteridae

Casey, 1916

Figs 2 , 4 ; Map 2

Phelister brevistriatus Casey, 1916: 233.

##### Type material.

**Holotype** of undetermined sex: “Tucson, Arizon, Wickham” / “Casey bequest 1925” / “TYPE USNM 38452” / “brevistriatus Csy.”, examined in 2011 (USNM).

##### Description.

Length: 1.81–2.29 mm (avg. 2.03 mm); width: 1.50–1.89 mm (avg. 1.69 mm). Body uniformly dark rufescent to piceous, elongate oval, widest just behind midline; frons finely but distinctly punctulate; supraorbital stria complete, fine across vertex; frontal stria well impressed along inner margin of eyes, continuing mediad above epistoma, but variably interrupted, often in a dense, linear field of punctures along frontal midline, ends generally curved upward, rarely complete and simple; labrum transverse, at most weakly emarginate apically; mandibles both with strong tooth along incisor margin; pronotum with sides subparallel at bases, weakly curved inward to front, disk very finely punctate at middle but rather coarsely so in lateral thirds; prescutellar impression weak, generally present; lateral and anterior marginal striae continuous, the anterior diverging slightly from the margin and crenulate; lateral submarginal striae absent; elytra with single, complete epipleural striae; outer subhumeral stria present in apical half only; inner subhumeral stria absent; dorsal elytral striae 1–4 complete, 5^th^ present in apical half and represented by a basal puncture, sutural stria slightly longer than fifth; propygidium almost uniformly coarsely punctate, punctures separated by less than their widths; pygidium much more finely and sparsely punctate; prosternal lobe shape evenly rounded, with more or less complete marginal stria; prosternal keel striae separate basally, converging between coxae, thence diverging weakly, usually connected by anterior arch, males with striae more widely separated, and punctures between striae slightly denser and more conspicuous; anterior mesoventral margin weakly produced; marginal mesoventral stria complete, smooth; mesometaventral stria more distinctly crenulate, arching anteriad to middle of mesoventrite; metaventral disk impunctate, lateral stria nearly complete to inner corner of metacoxa; postmesocoxal stria extending posterolaterad, wavering, becoming fragmented posteriad, ending well short of metepisternum; abdominal ventrite 1 with complete inner and fragmentary outer lateral striae, disk impunctate at middle, increasingly punctate to sides; protibia with apex obliquely truncate, outer margin weakly rounded, bearing 6–7 prominent, marginal spines; meso- and metatibiae weakly expanded to apex, mesotibiae with ca. six marginal spines, more prominent toward apex, metatibia with distinct spines along apical third of margin; aedeagus with distinct ventral process near middle, tegmen in dorsal view gradually widened toward apex, apically abruptly narrowed, with short, rather wide apical emargination; median lobe with long, basal apodemes distinctly thicker toward gonopore, nearly as long as tegmen.

**Figure 4.**
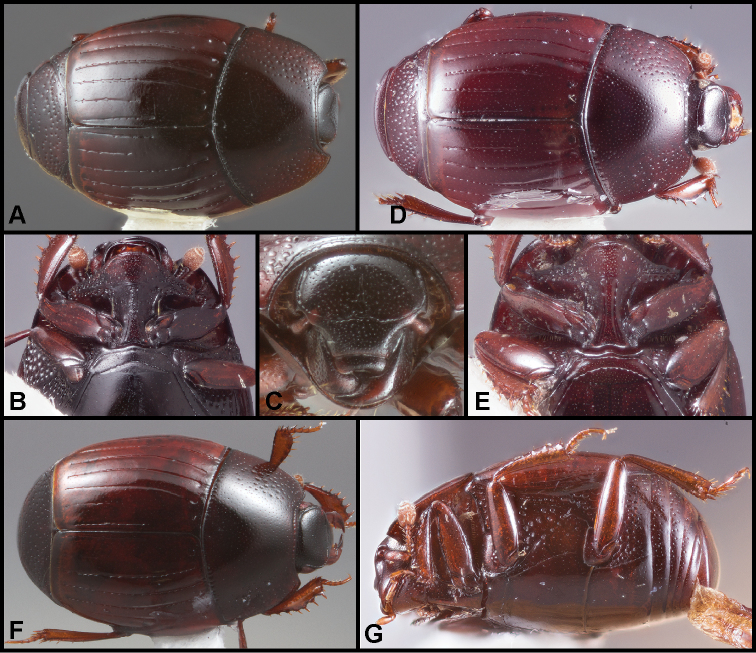
**A–C***Phelisterbrevistriatus* Casey: **A** Dorsal habitus **B** Ventral view showing prosternal and meso- and metaventral striae **C** Frontal view showing median portion of frontal stria **D–E***Phelistersonorae* sp. nov.: **D** Dorsal habitus **E** Ventral view showing prosternal and meso- and metaventral striae **F–G***Phelisterwarneri* sp. nov. **F** Dorsal habitus **G** Lateral view showing fine meso- and metaventral striae and slender tibiae with reduced lateral spines.

##### Remarks.

Of the *Phelister* species occurring in the southwestern United States and northwestern Mexico, this species can generally be recognized by its frontal stria, which, while usually complete, tends to connect to a series of median longitudinal frontal punctures. These are not always present, however. It seems to be closely related to the two following species, both of which have distinctive characters of their own. *Phelistersonorae* has modified protarsal claws (perhaps in the male only), while *Phelisterwarneri* has more finely impressed dorsal striae, and more finely spinose middle and hind tibiae.

##### Biology.

Label data reveal varied habits for this species. Many specimens were collected in cow dung. A few specimens were taken directly from kangaroo rat (*Dipodomys* Gray) burrows, and many others were collected using black pitfall traps in the vicinity of *Dipodomys* burrows, so facultative mammal inquilinism appears likely. Several specimens were also taken from the debris piles of leafcutter ants (*Attamexicana* (Smith) and *Atta* sp.)

##### Distribution.

This species ranges from the southwestern United States through Mexico into northern Central America. **Records: EL SALVADOR**: San Salvador, **MEXICO**: Chiapas, Colima, Hidalgo, Jalisco, Oaxaca, Puebla, San Luis Potosí, Sinaloa, Sonora; **USA: Arizona**: Cochise, Gila, Maricopa, Pima, Pinal, Santa Cruz; **California**: Imperial, Riverside; **New Mexico**: Hidalgo.

#### Phelister sonorae sp. nov.

Taxon classificationAnimaliaColeopteraHisteridae

http://zoobank.org/53CAD355-2FEF-48FE-84B4-1573E869F0BA

Figs 2 , 4 ; Map 2

##### Type material.

**Holotype male**: “MEX., Sonora Don [sp?], 14 Aug, 1957, David Lauck, small mud pond”/”Phelisteraffinis det S. Mazur”/”Caterino/Tishechkin Exosternini voucher EXO-03591”; deposited in USNM.

##### Diagnostic description.

Length: 1.97 mm; width: 1.58 mm. This species is externally very difficult to distinguish from *P.brevistriatus*, especially given the variability and wide geographic distribution of that species (within which *P.sonorae* occurs). With only a single specimen of this species, we can confidently cite only: male protarsal claws strongly bent at base, straight in apical three-fourths; frons narrower, with frontal stria less strongly impressed, weakly interrupted in denser frontal punctation; ground punctation of pronotal disk coarser and deeper; 5^th^ dorsal elytral stria nearly complete, continued between stria and basal puncture by a weak crease; male prosternal keel striae not as widely separated, and surface between striae not very conspicuously punctate; aedeagus lacking distinct ventral process, tegmen narrow, only slightly widened at middle, apices acute, separated, apical emargination incised to middle of tegmen; median lobe with short, basal apodemes distinctly thicker toward gonopore, only ca. one-third as long as tegmen.

##### Remarks.

As mentioned in the description, externally this species mostly falls within the range of variation for most characters of *P.brevistriatus*. Aside from the very distinctive aedeagus, and unusual, probably sex-limited, bent protarsal claw, the only distinguishing feature is the longer 5^th^ dorsal elytral stria. More material of this species will be necessary to confirm these differences, and hopefully to support the consistency of some other minor characters.

##### Etymology.

We name the species for the state and region of its origin.

##### Biology.

Nothing is known of the biology of this species.

##### Distribution.

This species is only known from Sonora, Mexico.

#### Phelister warneri sp. nov.

Taxon classificationAnimaliaColeopteraHisteridae

http://zoobank.org/3A7CF0EF-94DC-4ADE-ADDF-8A3B6B839743

Figs 2 , 4 ; Map 2

##### Type material.

**Holotype male**: “USA: AZ: Cochise Co., Birch Rd., 4.1 mi. E of Hwy 191, 31°58'43"N, 109°46'4"W; vii.17-29.2011; black cup pitfalls; W.BWarner” / “Caterino/Tishechkin Exosternini Voucher EXO-03588”; deposited in ASUC; Paratypes (11): 4: same data as type; 2: same locality but vii.29-viii.14.2011; 3: same locality but vii.30-ix.5.2012; 1: same locality but vii.14-28.2011; 1: AZ: Cochise Co. Hwy 186 at Blue Sky Rd.; 32°12'52"N, 109°46'54"W, vii.17-29.2011, ex black cup barrier pitfall; W.B. Warner; 1: AZ: Cochise Co., 1.5 mi S. jct. Hwys 191 and 181; 31°51'44"N, 109°41'59"W; vii.14-28.2011; black cup barrier pitfall; W.B. Warner. Deposited in MSCC, AKTC, FMNH, and WBWC. Additional material: TX: Brewster Co., Marathon Iron Mt. Ranch, v.12.1976, R. Gordon, in burrow of *Cynomysludovicianus* (USNM).

##### Diagnostic description

Length: 1.73–2.09 mm (avg. 1.88 mm); width: 1.46–1.69 mm (avg. 1.57 mm). This species is externally very difficult to distinguish from *P.brevistriatus*, as well as *P.sonorae*. The following characters should be sufficient to distinguish *P.warneri*: body distinctly rufescent, with the elytra (except for a linear area right along the suture) vaguely lighter/brighter red than most of the rest of the body, the lateral regions of the pronotum sometimes appearing similarly lighter; frontal stria often interrupted at sides as well as medially, rarely obsolete across the front; frontal disk usually with enlarged median punctures, but not organized into a linear cluster as they are in *P.brevistriatus*; ground punctation of pronotum more distinct, grading more gradually into denser lateral pronotal punctures; male protarsal claws ‘normal’, curved, not bent at base (distinct from *P.sonorae)*; elytral striae shallowly and rather finely impressed, 5^th^ dorsal elytral stria confined to posterior third of elytron, typically fragmented, rarely entirely obsolete; meso- and metatibiae narrower, slightly more elongate, with marginal spines fewer in number and size; 1^st^ abdominal ventrite with little or no vestige of outer lateral stria; aedeagus with short basal piece ca. one-fifth total aedeagus length; tegmen rather narrow, dorsoventrally flattened, with small ventral process, tegmen sides subparallel, undulating, apices slightly convergent but separate, apical emargination broad and deep, ca. one-third tegmen length; median lobe ca. one-third tegmen length, with simple proximal apodemes.

##### Etymology.

In naming this species for Mr. Bill Warner, we are pleased to recognize his many contributions to our knowledge of histerid biology, taxonomy, and distribution. His efforts led to the discovery of this species, and many others.

##### Biology.

The type series of this species was collected in the same place, and even in the same black cup pitfall traps, as numerous specimens of *P.brevistriatus*. Black cup pitfalls evidently often attract mammal nest inquilines (the cup imitates a burrow entrance; WB Warner, pers. comm.), and we suggest that *P.warneri* is a specialized inquiline. The single specimen from Texas was collected in a burrow of black-tailed prairie dog (*Cynomysludovicianus*), supporting this assertion. This potential host does extend into southeastern Arizona.

##### Distribution.

This species is mainly known from a single locality in southeastern Arizona. An additional male (which we have dissected) from Brewster Co., Texas, however, conforms in all respects to the diagnosis above. So, the species must be more widespread.

#### Phelister puncticollis

Taxon classificationAnimaliaColeopteraHisteridae

Hinton, 1935

Figs 2 , 5 ; Map 3

Phelister puncticollis Hinton, 1935c: 64.

##### Type material.

**Holotype** of undetermined sex: “H.H. Smith, S. Amer” / “Monte Alegre” [Pará] / “Phelister puncticollis Type Hntn.” / “G. Lewis Coll. B.M.1926-369”, NHMUK.

##### Diagnostic description.

Length: 1.54–1.65 mm (avg. 1.59 mm); width: 1.30–1.38 mm (avg. 1.29 mm). Body elongate oval, dark rufescent, with elytra lighter toward their apices; frons depressed along midline, disk strongly punctate; frontal striae obsolete between antennal insertions; labrum short, weakly emarginate; both mandibles with distinct median tooth, that of right mandible small; entire pronotal disk almost uniformly punctate, only slightly less dense at middle; prescutellar impression distinct, though somewhat obscured by discal and posterior marginal punctures; marginal pronotal stria complete along lateral and anterior margins, crenulate anteriorly; lateral submarginal stria complete at sides, curving mediad at front, ending freely behind eye; elytra with complete outer subhumeral stria, inner subhumeral stria absent; dorsal elytral striae 1–5 complete, sutural stria obsolete in basal one-third, all striae distinctly crenulate; propygidium almost uniformly punctate, with small round punctures separated by approximately their diameters; pygidial punctures smaller and sparser, fading to indistinct at apex; prosternal lobe short, with fine, complete marginal stria; prosternal keel with striae united anteriorly to form a triangle, the male’s more densely punctate within; mesoventrite weakly projecting at middle; marginal mesoventral stria complete, evenly arched anteriad between inner corners of mesocoxae; mesometaventral stria arched strongly forward to mesoventral midpoint, extended by inner mesoventral stria to metacoxa; metaventral disk with distinct ground punctation and coarser punctures along most of posterior third; 1^st^ abdominal ventrite with complete inner lateral stria and fragments of outer lateral stria; protibia with lateral margin strongly rounded, with 6–7 marginal spines, apex obliquely truncate, with two small apical spurs; protarsal claws somewhat unevenly curved, slightly bent at base; meso- and metatibiae evenly widened to apices, with few weak marginal spines confined to apical halves; basal piece of aedeagus ca. one-fourth total aedeagus length; tegmen narrow at base, widened toward narrowly rounded apex, apical emargination narrow, incised ca. one-fourth of tegmen length, ventral process absent; median lobe with long proximal apodemes, evenly differentiated into thicker and thinner portions.

**Figure 5.**
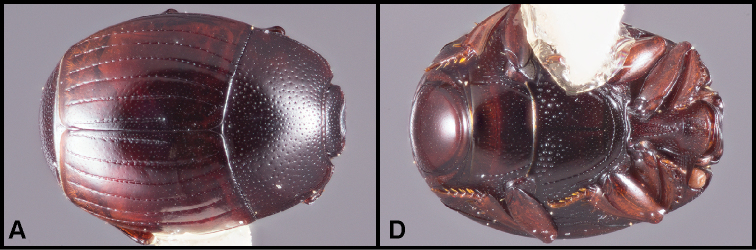
**A-B***Phelisterpuncticollis* Hinton: **A** Dorsal habitus **B** Ventral view.

##### Remarks.

This species is similar to *P.bryanti* described below, but differs in the presence of a submarginal pronotal stria, abbreviated, united male prosternal striae, and the presence of punctures on the metaventrite in front of the metacoxae. The aedeagus of *P.puncticollis* is narrow and evenly rounded to the apex, whereas that of *P.bryanti* is abruptly narrowed.

##### Biology.

Nothing is known of the biology of this species.

##### Distribution.

We have only seen specimens from Pará state, Brazil, including the types, and only three additional specimens, from Belém and Benevides. One specimen was collected in the nest of the fire ant *Solenopsissaevissima* (Smith).

**Map 3.**
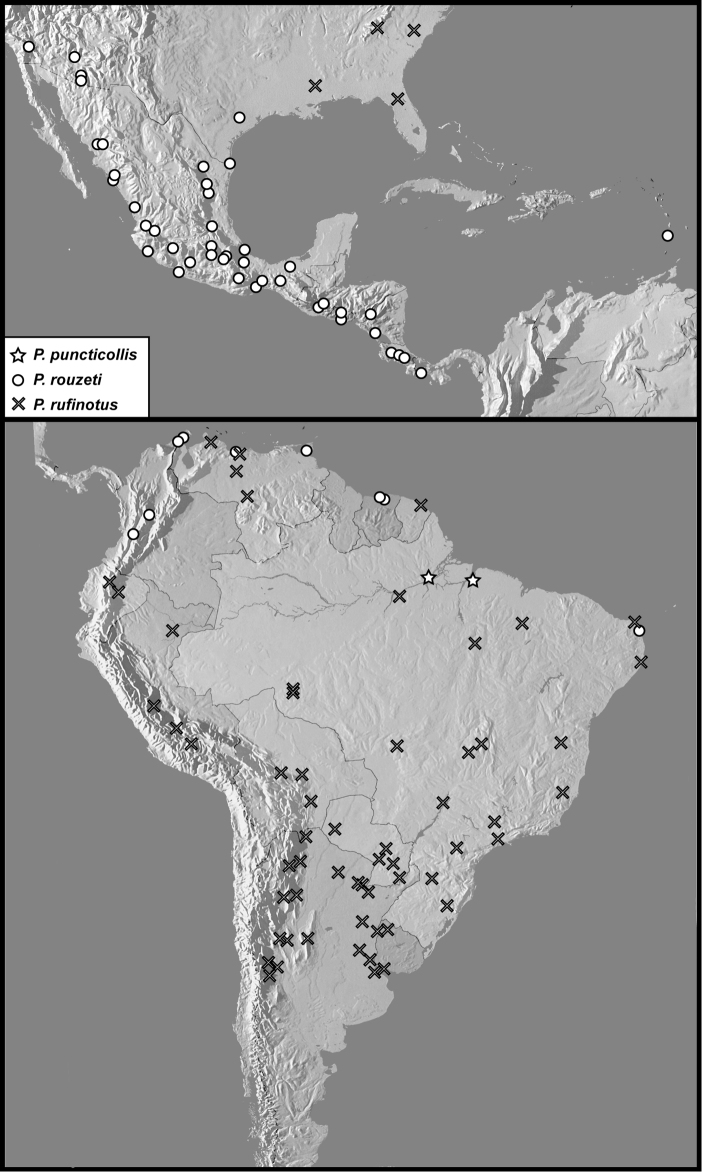
Collecting records for *Phelisterpuncticollis* (stars), *P.rouzeti* (circles), and *P.rufinotus* (diagonal crosses).

#### Phelister subrotundus

Taxon classificationAnimaliaColeopteraHisteridae

(Say, 1825)

Figs 2 , 6 ; Map 4

Hister subrotundus Say, 1825: 39.
Phelister subrotundus : Marseul 1853: 487.
Phelister rubricatus Lewis, 1908: 158; Mazur 1997: 29.
Phelister subrotundus var. sayi Carnochan, 1915: 213; Mazur 1997: 29.
Phelister subrotundus var. frosti Carnochan, 1915: 213; Mazur 1997: 29.
Phelister carnochani Casey, 1916: 291; Mazur 1984: 285.
Phelister contractus Casey, 1916: 230, syn nov. (previously synonymized with P.affinis by Mazur (1997), in error).
Phelister subrotundatus : Mazur 1984: 286 (misspelling).

##### Type material.

**Neotype** of *Histersubrotundus* Say, hereby designated: [pale pinkish round disk] / “892” / “NEOTYPE Hister subrotundus Say Desg. Caterino & Tishechkin, 2011”, MCZC. This common, widespread, and somewhat variable Nearctic species needs to be represented by a physical type so as to establish the identity of *P.subrotundus*, in the event that later work reveals it to represent multiple species.

Types of synonyms. **Lectotype** of *Phelisterrubricatus* Lewis hereby designated: “Type” [red bordered disk] / “Dane Co., Wis. IX.30.99” / “G.Lewis Coll. B.M.1926-369” / “Phelister rubricatus Lewis Type” / “LECTOTYPE Phelister rubricatus Lewis M.S. Caterino and A.K. Tishechkin des. 2010”, NHMUK; Paralectotype: “Eddyville, IA” (interpreted by G. Lewis [1908] as Eddyville, Ja. [sic]) / “G.Lewis Coll. B.M.1926-369” / “Phelister rubricatus Lew. Cotype” / “PARALECTOTYPE Phelister rubricatus Lewis M.S. Caterino and A.K. Tishechkin des. 2010”, NHMUK. **Holotypes** of Carnochan’s ‘varieties’, P.subrotundusvar.sayi and P.subrotundusvar.frosti, are in MCZ (#26012 and 26013, respectively.) **Lectotype** of *Phelistercarnochani* Casey hereby designated: “Alab 2289” / “Casey bequest 1925” / “TYPE USNM 38448” / “carnochani Csy. sayi Csy nec Carn.” / “LECTOTYPE Phelister carnochani Casey M.S. Caterino and A.K. Tishechkin des. 2019, USNM. **Holotype** of *Phelistercontractus* Casey: “Lee Co Tex” / “Casey bequest 1925” / “TYPE USNM 38447” / “contractus Csy.”, USNM. This species was previously synonymized, in error, with *Phelisteraffinis* by Mazur (1997). Wenzel (unpub. notes) agrees with our assessment.

##### Diagnostic description.

Length: 1.54–1.85 mm (avg. 1.69 mm); width: 1.30–1.62 mm (avg. 1.52 mm). Body elongate-oval, widest behind humeri, mostly piceous, posterolateral corners of elytra and legs generally reddish; entire dorsum finely punctulate, the pronotum more densely so than the elytra; frons finely punctulate, impressed along midline, supraorbital stria complete, frontal stria interrupted at middle, slightly sinuate at sides; labrum wide, weakly emarginate apically; both mandibles with strong tooth on inner edges; pronotum with more or less complete lateral submarginal stria incurved and crenulate anteriorly, ending freely, and diverging slightly from pronotal margin posteriorly, where it is weakly abbreviated; pronotal disk with larger punctures interspersed with finer punctures along lateral thirds; elytron with single, complete epipleural stria, outer subhumeral stria present in apical third, inner subhumeral stria absent, dorsal striae 1–5 complete, sutural stria obsolete in basal third; propygidium with distinct secondary punctures decreasing in density posteriad; pygidium more finely punctate; prosternal keel with two complete striae, weakly convergent and free anteriorly, usually united along basal margin of keel; male prosternal keel with coarser and denser punctures, the striae often more widely separated and more nearly parallel; mesoventral marginal stria complete, weakly crenulate, close to anterior mesoventral margin, often with corresponding median ‘point’, continued at sides by postmesocoxal stria which ends freely midway between the meso- and metacoxae; mesometaventral stria complete, crenulate at middle, arched anteriad distinctly onto mesoventrite (with weakly parallel median ‘point’ to mesoventral stria), curving posteriad to near inner corner of metacoxa; first abdominal ventrite with complete inner lateral stria and abbreviated outer lateral stria; protibia with apex obliquely truncate, outer margin weakly rounded, bearing ca. six evenly spaced marginal spines; meso- and metatibiae weakly expanded to apex, mesotibia with ca. five marginal spines, more prominent toward apex, metatibia with distinct spines confined to apical fourth. Aedeagus with basal piece ca. one-fourth total length; tegmen widened toward apex, apex evenly rounded, with shallow apical emargination; median lobe ca. two-thirds tegmen length, with differentiated basal and distal proximal apodemes.

**Figure 6.**
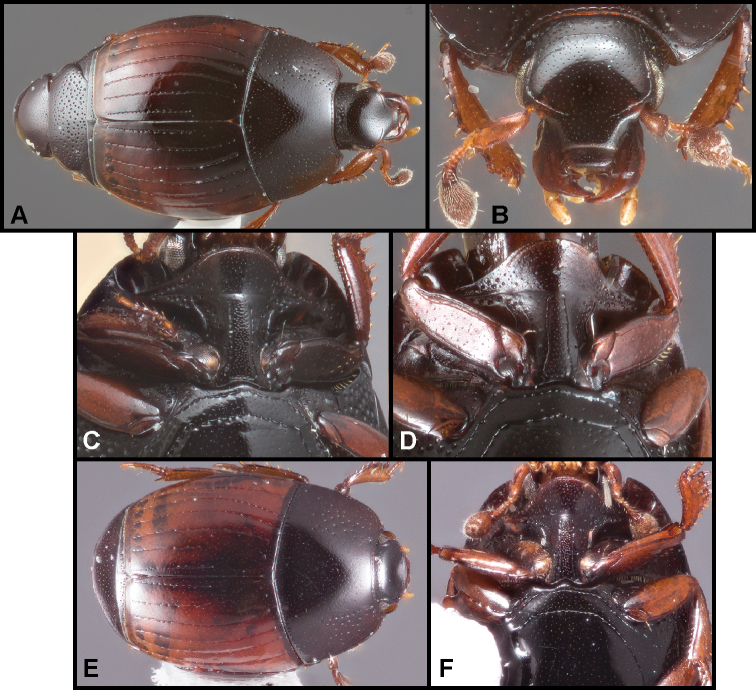
**A–D***Phelistersubrotundus* (Say): **A** Dorsal habitus **B** Frontal view showing frontal striae **C** Prosternum and mesoventrite of male **D** Prosternum and mesoventrite of female **E–F***Phelisterrouzeti* (Fairmaire) **E** Dorsal habitus **F** Ventral view of prosternum of male and meso- and metaventrites showing striae.

##### Remarks.

Among species occurring in the United States, *P.subrotundus* is easily separated by the following character states: elytra reddish posterolaterally; frons depressed, with frontal stria interrupted; submarginal pronotal stria present, more or less complete, curved mediad anteriorly and diverging from margin posteriorly; prosternal striae converging anteriorly to nearly parallel, intervening punctures denser in male; elytral stria 1–5 complete. Below we refer to this species and following four (*P.rufinotus*, *P.thiemei*, *P.rouzeti*, and *P.parecis*) informally as the *P.rufinotus* complex, and their close relationship is supported by phylogenetic analyses to date.

##### Biology.

The species has diverse and general habits, having been collected very commonly in dung, as well as in decaying vegetation, leaf litter, seaweed on the beach, in pocket gopher (*Geomys*) burrows, and even with a few ant species (in the genera *Aphaenogaster* Mayr and *Formica* L.). A few of the specimens from pocket gopher burrows, including one from Arkansas and four from Georgia, are unusually small and have a posteriorly abbreviated sublateral pronotal stria. Males from both localities were dissected and do not differ in any obvious way from others of the species, so we have not considered them distinct. Further study should more carefully address this possibility with molecular data. Interestingly, a long series from *Geomysbursarius* (Shaw) burrows from St Clair Co, Illinois, do not exhibit these differences.

##### Distribution.

This is the most abundant and widespread *Phelister* species in North America, occurring from southeastern Canada to Florida, west to South Dakota and Arizona. We have not seen any records from Mexico, but it occurs in several US border counties, and must occur south of the border as well. **Records: CANADA: Ontario; Quebec; USA: Alabama**: Blount, Greene, Marion; **Arizona**: Pima, Santa Cruz; **Arkansas**: Calhoun, Lafayette, Lee, Little River, Scott, Washington; **Delaware**: Kent, Sussex; **District of Columbia; Florida**: Alachua; **Georgia**: Baker, Burke, Clarke, Dodge, Lamar, Peach, Talbot, Thomas, Wheeler; **Illinois**: Champaign, Coles, Cook, Grundy, Iroquois, Jackson, Jo Daviess, Kane, Kankakee, Lake, Mason, McHenry, Pope, St. Clair, Will; **Indiana**: Boone, Brown, Franklin, Jasper, Lake, LaPorte, Laurel, Lawrence, Monroe, Newton, Parke, Porter, Starke, Tippecanoe, Vanderburgh; **Iowa**: Butler, Cedar, Dickinson, Dubuque, Johnson, Marshall, Monroe, Muscatine, Plymouth, Story, Warren; **Kansas**: Bourbon, Decatur, Doniphan, Douglas, Jefferson, Kiowa, Labette, Leavenworth, Miami, Montgomery, Norton, Pottowatomie, Rawlins, Riley, Sedgwick, Shawnee; **Kentucky**: Bell, Henderson; **Louisiana**: Bienville, Orleans, St. Charles, West Feliciana; **Maine**: Oxford; **Maryland**: Anne Arundel, Calvert, Charles, Garret, Montgomery, Prince George’s, Somerset, Talbot, Washington; **Massachusetts**: Hampden, Hampshire, Middlesex, Plymouth; **Michigan**: Ingham, Kalamazoo, Macomb, Washtenaw, Wayne; **Minnesota**: Brown; **Mississippi**: Harrison, Oktibbeha, Pontotoc; **Missouri**: Boone, Marion, Mississippi, Pike, Saint Louis, Scott, Taney, Washington, **Nebraska**: Clay, Lancaster, Lincoln, Saunders; **New Jersey**: Bergen, Camden, Cape May, Gloucester, Hudson, Morris, Passaic, Sussex, Union; **New Hampshire**: Grafton; **New Mexico**: Hidalgo; **New York**: Cattaraugus, Kings, Monroe, Nassau, Onondaga, Orange, Richmond, Seneca, St. Lawrence, Suffolk, Tompkins; **North Carolina**: Buncombe, Duplin, Edgecombe, Jackson, New Hanover, Swain, Wake; **North Dakota**: Cass, Richland; **Ohio**: Ashland, Clermont, Cuyahoga, Franklin, Hocking, Lucas, Medina, Ross, Scioto, Summit, Wayne; **Oklahoma**: Cleveland, Comanche, Craig, Grant, Hughes, Latimer, Marshall, McCurtain, Payne, Sequoyah, Woods; **Pennsylvania**: Bradford, Cambria, Chester, Dauphin, Fulton, Luzerne, Monroe, Montgomery, Perry, Philadelphia; **South Carolina**: Anderson, Bamberg, Charleston, Dorchester, Florence, Pickens, Richland; **South Dakota**: Jackson, Lawrence, Minnehaha, Pennington, Yankton; **Tennessee**: Benton, Blount, Davidson, Hamilton, Montgomery, Morgan, Sevier, Wilson; **Texas**: Brazos, Colorado, Dallas, Denton, Duval, Erath, Goliad, Hidalgo, Jim Hogg, Montague, Val Verde; **Virginia**: Arlington, Fairfax, Nansemond, Nelson, Spotsylvania; **West Virginia**: Berkeley, Braxton, Grant, Greenbrier, Mason, Mineral, Preston, Putnam, Wayne; **Wisconsin**: Crawford, Dane, Iowa, Jefferson, Kenosha, Lafayette, Richland, Sauk, Shawano, Walworth, Waupaca.

**Map 4.**
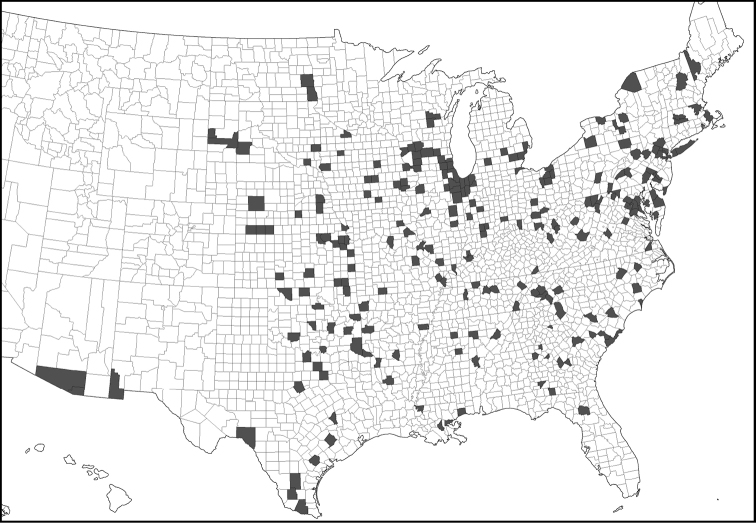
United States county map shading counties with records of *Phelistersubrotundus*. Not shown are records from southern Ontario and Quebec, Canada (see Bousquet & Laplante 2006). No records have been found from Mexico.

#### Phelister rouzeti

Taxon classificationAnimaliaColeopteraHisteridae

(Fairmaire, 1850)

Figs 2 , 6 ; Map 3

Paromalus rouzeti Fairmaire, 1850: 421.
Phelister rouzeti ; Marseul, 1853: 488.
Phelister fairmairei Marseul, 1861: 172; **syn. nov.** (previously synonymized with P.rufinotus, Mazur, 1997, in error)
Phelister wickhami Casey, 1916: 231, **syn. nov.**
Phelister pimalis Casey, 1916: 233; Mazur 1997: 29.
Phelister aztecanus Casey, 1916: 233; Mazur 1997: 29.

##### Type material.

**Lectotype** hereby designated: “Bondy fourmis Rouzet” / “certe Mexicanum” [handwritten, perhaps by Marseul] / “LECTOTYPE Paromalus rouzeti Fairmaire M.S. Caterino & A.K. Tishechkin des. 2010”, MNHN.

Types of synonyms. **Lectotype***Phelisterfairmairei* Marseul hereby designated: “Caracas Sallé” / “G.Lewis Coll. B.M.1926-369” / “LECTOTYPE Phelister fairmairei Marseul M.S. Caterino and A.K. Tishechkin des. 2010”, NHMUK. One paralectotype, same data as lectotype, NHMUK. **Lectotype** of *Phelisterwickhami* Casey hereby designated: “Tepehuanes Dgo. Mex. Wickham” / “Casey bequest 1925” / “TYPE USNM 38449” / “wickhami Csy.” / “LECTOTYPE Phelister wickhami Casey M.S. Caterino and A.K. Tishechkin des. 2010.”, USNM. **Lectotype** of *Phelisterpimalis* Casey hereby designated: “Ari” / “Casey bequest 1925” / “TYPE USNM 38454” / “pimalis Csy.” / “LECTOTYPE Phelister pimalis Casey M.S. Caterino and A.K. Tishechkin des. 2019.”, USNM. One paralectotype with same data, USNM. **Lectotype** of *Phelisteraztecanus* Casey hereby designated: “San Angel D.F. Mex” / “Casey bequest 1925” / “TYPE USNM 38455” / “aztecanus Csy.” / “LECTOTYPE Phelister aztecanus Casey M.S. Caterino and A.K. Tishechkin des. 2019.”, USNM. One paralectotype with same data, USNM.

##### Diagnostic description.

Length: 1.38–1.77 mm (avg. 1.57 mm); width: 1.10–1.46 mm (avg. 1.32 mm). Body elongate-oval, widest behind humeri; body color varied, common with much of elytra (posterolaterally) reddish, more rarely entirely piceous dorsally, legs typically golden reddish, though darker in piceous specimens; elytra and pronotum very finely punctulate; frons finely punctulate, impressed along midline, supraorbital stria complete, frontal stria interrupted at middle, slightly sinuate at sides; labrum wide, not or only weakly emarginate apically; both mandibles with strong tooth on inner edges; pronotum with more or less complete lateral submarginal stria incurved and crenulate anteriorly, ending freely, and diverging slightly from pronotal margin posteriorly, where it is often weakly abbreviated; pronotal disk with distinct, elongate secondary punctures along lateral thirds; base of pronotum with cluster of larger punctures in front of suture; elytron with single, complete epipleural stria, outer subhumeral stria present in apical third, inner subhumeral stria absent, dorsal striae 1–4 complete, fifth stria usually present in just over apical half, rarely complete, sutural stria present in apical two-thirds; propygidium with distinct secondary punctures decreasing in density posteriad; pygidium more finely punctate; prosternal keel with two complete striae, weakly convergent to subparallel, occasionally sinuate, free anteriorly, usually united along basal margin of keel; male prosternal keel with coarser and denser punctures, the males’ striae often more widely separated; mesoventral marginal stria complete, continued at sides by sinuate postmesocoxal stria which ends freely midway between the meso- and metacoxae; mesometaventral stria complete, crenulate at middle, arched anteriad onto mesoventrite at middle, continued posteriad to near inner third of metacoxal margin; first abdominal ventrite with complete inner lateral stria and abbreviated outer lateral stria; protibia with apex obliquely truncate, outer margin weakly rounded, bearing ca. five evenly spaced marginal spines plus a larger apical marginal spine separated from others by a greater gap; meso- and metatibiae weakly expanded to apex, mesotibia with ca. five marginal spines, more prominent toward apex, metatibia with distinct spines mainly in apical half. Aedeagus with basal piece ca. one-fifth total length; tegmen simple, widest near middle, converging to apex, apex distinctly emarginate; median lobe ca. two-thirds tegmen length, with differentiated basal and distal proximal apodemes.

##### Remarks.

Although its distribution is rather broad, *P.rouzeti* seems to be primarily native to western Mexico and southwestern North America. Its distribution only barely overlaps with that of *P.subrotundus*, its closest relative, from which it can be separated by its more elongate body form, usually abbreviated 5^th^ elytral stria, and its narrower, more tapered aedeagus. Where it overlaps in distribution with *P.rufinotus*, in northern South America, the complete submarginal pronotal stria of *P.rouzeti* will distinguish them. Although the species was originally described from France (Bondy, northeast of downtown Paris), it has never since been recorded in the country, despite dedicated attempts to recollect it (Auzat 1925; M Secq, pers. comm.). While an introduction followed by extirpation cannot be ruled out, it seems more likely that the original specimen was mislabeled, and that the species has never inhabited Europe.

##### Biology.

Label data indicate varied habits for this species, with records from fire-scorched *Yucca*, rotten *Opuntia*, under bark of *Celtis* L., on a fermenting orange, in cow, dog, and horse dung, and in sifted riparian ‘dirt’.

##### Distribution.

This widespread species occurs from the southwestern and south-central United States, through Mexico and Central America into northern South America. The species also occurs on several islands in the West Indies. As a common cow dung associate, its range has likely expanded in post-Columbian times. **Records: BRAZIL**: Rio Grande do Norte; **COLOMBIA**: La Guajira, Magdalena, Palmira, Tolima; **COSTA RICA**: Guanacaste, Heredia; **EL SALVADOR**: La Libertad, San Salvador,; **GUATEMALA**: Escuintla; **MEXICO**: Chiapas, Colima, Distrito Federal, Guerrero, Jalisco, México, Michoacán, Morelos, Nayarit, Nuevo León, Oaxaca, Puebla, San Luis Potosí, Sinaloa, Sonora, Tabasco, Tamaulipas, Veracruz; **NICARAGUA**: Granada, Madriz; **PANAMA**: Chiriquí; **SAINT VINCENT & GRENADINES**: Saint Vincent; **SURINAME**: Pará, Saramacca; **TRINIDAD & TOBAGO**: Trinidad; **VENEZUELA**: Aragua; **USA: Arizona**: Florida, Maricopa, Pima, Santa Cruz; **California**: Riverside, **Texas**: Cameron, Colorado.

#### Phelister rufinotus

Taxon classificationAnimaliaColeopteraHisteridae

Marseul, 1861

Figs 2 , 7 ; Map 3

Phelister rufinotus Marseul, 1861: 170.
Epierus marseulii Kirsch 1873: 136, syn. nov.

##### Type material.

Type locality: “Bresil, Rio-Janeiro”. We have been unable to find any specimens that we believe to validly represent syntypes of *Phelisterrufinotus*. Marseul specimens (with circular, green, handwritten labels) are present in MNHN and in NHMUK, but all of these represent other localities and appear to have been collected later ([18]‘63’ and [18]’68) than the species was described. A couple of specimens in the NHMUK are labeled with variations on Rio de Janeiro, but none as typical for Marseul types, and lacking collection dates, it’s impossible to tell if these might have been extant in 1861. We considered designating a Neotype from among the later Marseul-identified specimens. However, we feel that the species is now adequately characterized, and that this would not serve a critical need.

Types of synonyms. **Lectotype** of *Epierusmarseulii* Kirsch, hereby designated: “Pozuzu M. Kirsch” / “Statl. Museum für Tierkunde, Dresden” / “Epierus marseulii” / “Phelister rufinotus Mars. n. syn.” / “LECTOTYPE Epierus marseuli [sic] Kirsch M.S. Caterino and A.K. Tishechkin des. 2010”, MTD.

##### Diagnostic description.

Length: 1.46–1.69 mm (avg. 1.56 mm); width: 1.30–1.50 mm (avg. 1.34 mm). Body elongate-oval, widest just behind humeri; body piceous to rufescent, most of elytra and legs usually contrastingly reddish (elytra sometimes nearly black); dorsum very finely punctulate, the pronotum more densely so than the elytra, especially in the outer thirds; frons finely punctulate, impressed along midline, supraorbital stria complete, frontal stria interrupted at middle, inner ends pointing toward epistoma; labrum wide, weakly emarginate apically; both mandibles with distinct tooth on inner edges; pronotal lateral submarginal stria abbreviated, present in anterior half only; pronotal disk with vague antescutellar impression, with crenulations along posterior margin; elytron with single, complete epipleural stria, outer subhumeral stria present in apical half, inner subhumeral stria absent, dorsal striae 1–4 complete, fifth variable, but at least weakened in basal third if not obsolete, sutural stria obsolete in basal third; propygidium with sparse secondary punctures decreasing in density posteriad; pygidium with secondary punctures fewer and finer, diminishing to apex; prosternal keel with two complete striae, weakly sinuate, subparallel at base, slightly converging toward apex, free anteriorly; male prosternal keel with coarser and denser punctures, the striae often more widely separated; mesoventral marginal stria complete, smooth, continued at sides by postmesocoxal stria which ends freely near side of mesoventrite; mesometaventral stria complete, very weakly crenulate, arched anteriad nearly to midline of mesoventrite extended posteriad by lateral metaventral stria toward middle of metacoxa, ending short of it; first abdominal ventrite with complete inner lateral stria and abbreviated outer lateral stria; protibia rather narrow, with apex obliquely truncate, outer margin weakly rounded, bearing ca. five evenly spaced marginal spines, the spine of the apical corner larger and slightly disjunct; meso- and metatibiae weakly expanded to apex, mesotibia with ca. five marginal spines, more prominent toward apex, metatibia with distinct spines confined to apical third. Aedeagus with basal piece ca. one-fourth total length; tegmen widened toward apex, spoon-shaped, apex rounded, with narrow apical emargination; median lobe ca. two-thirds tegmen length, with differentiated proximal and shorter distal proximal apodemes.

**Figure 7.**
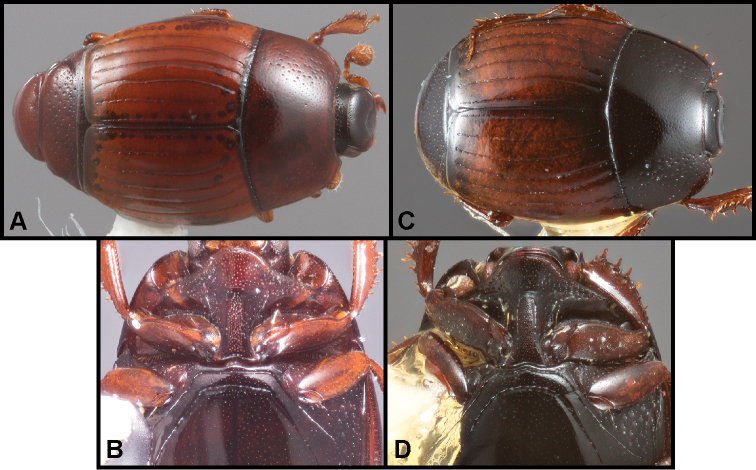
**A–B***Phelisterrufinotus* Marseul: **A** Dorsal habitus **B** Prosternum and mesoventrite of male. **C–D***Phelisterthiemei* Schmidt: **A** Dorsal habitus **B** Prosternum and mesoventrite of male.

##### Remarks.

This species is highly variable, but it can nearly always be recognized by its abbreviated submarginal pronotal stria. It is most often distinctly reddish on the elytra, but many all-black individuals have been seen, throughout the range. Its legs are nearly always distinctly golden in contrast to a piceous venter.

This species was previously synonymized with *P.fairmairei*Marseul 1861. We have studied that type and believe that *P.fairmairei* is instead identical to *P.rouzeti* (see above).

##### Biology.

Label data indicate broad ecological associations with records from cow dung, decaying vegetation, meat- and dung-baited pitfalls, and gopher tortoise droppings. The species also exhibits distinct tendencies toward facultative myrmecophily, with numerous records from *Acromyrmex* and *Solenopsis* Westwood, and even a few from *Eciton* Latreille (‘with prey’).

##### Distribution.

This species occurs most abundantly in southern South America, though numerous records also indicate that it occurs well into the tropics, with records in nearly every other country in the continent. There are several records from the Gulf Coast of the United States (Carolinas, Mississippi and Florida) that would seem likely to represent an introduction, given the lack of intervening records. This is another species that may well have expanded its distribution with the spread of cattle production. **Records: ARGENTINA**: Buenos Aires, Catamarca, Chaco, Cordoba, Corrientes, Entre Rios, La Rioja, Mendoza, Salta, San Juan, Santa Fe, Tucuman; **BOLIVIA**: Cochabamba, Santa Cruz; **BRAZIL**: Distrito Federal, Espírito Santo, Goias, Maranhão, Mato Grosso, Mato Grosso do Sul, Minas Gerais, Pará, Paraná, Pernambuco, Rio Grande do Norte, Rio Grande do Sul, Rondônia, Santa Catarina, São Paulo, Tocantins; **ECUADOR**: Napo, Pinchincha; **FRENCH GUIANA**: Cayenne; **PARAGUAY**: Boquerón, Caaguazú, Caazapá, Cordillera, Itapúa, San Pedro; **PERU**: Apurímac, Ayacucho, Junín, Loreto; **URUGUAY**: Colonia, Salto; **VENEZUELA**: Aragua, Bolívar, Falcón, Guárico; **USA: Florida**: Alachua; **Mississippi**: Jones; **North Carolina**: Jackson; **South Carolina**: Chesterfield.

#### Phelister thiemei

Taxon classificationAnimaliaColeopteraHisteridae

Schmidt, 1889

Figs 2 , 7 ; Map 5

Phelister thiemei Schmidt, 1889: 338.
Phelister stercoricola Bickhardt, 1909: 223, syn. nov.

##### Type material.

**Lectot**

##### ype,

hereby designated: “Matto grosso” / “Thiemei” / “Type” / “coll. J.Schmidt” / “Thiemei Schm.” / “LECTOTYPE Phelister thiemei Schmidt 1889, M.S. Caterino & A.K. Tishechkin des. 2010”, ZMHB.

Types of synonyms. **Lectotype** of *Phelisterstercoricola* Bickhardt hereby designated: “Montevideo, J. Tremoleras” / “Type” / “stercoricola Bickh.” / “LECTOTYPE Phelister stercoricola Bickhardt, 1909 M.S. Caterino & A.K. Tishechkin des. 2010”, ZMHB; five paralectotypes with same data, four in ZMHB, one in NHMUK.

##### Diagnostic description.

Length: 1.30–1.77 mm (avg. 1.53 mm); width: 1.06–1.50 mm (avg. 1.32 mm). This species is extremely similar to both *P.rufinotus* and *P.rouzeti*, differing principally in the following features: Body elongate-oval, widest behind humeri; nearly always distinctly bicolored, with much of elytra (posterolaterally) reddish, rarely entirely piceous dorsally; frontal stria complete to narrowly interrupted; lateral submarginal pronotal stria absent; dorsal elytral striae 1–4 complete, 5^th^ variable, complete to abbreviated from base; prosternal keel striae subparallel to sinuate; male prosternal keel with coarser and denser punctures; mesometaventral stria closer, almost subparallel to mesoventral stria; aedeagus with basal piece ca. one-fourth total length; tegmen simple, widest just beyond middle, subparallel in apical third, apex shallowly emarginate, tegmen in lateral view thickest toward apex; median lobe ca. two-thirds tegmen length, with differentiated basal and distal proximal apodemes.

##### Remarks.

We have characterized this species rather broadly. The typical form, from Mato Grosso, has a complete frontal stria and abbreviated 5^th^ dorsal stria. Considerable variation is observed in these characters from other areas, with the frontal stria more often interrupted elsewhere. Typical *P.stercoricola* (which we synonymize here) exemplifies this alternative, with an interrupted frontal stria and complete 5^th^ dorsal elytral stria. However, while there is some variation in genitalic shape over this range (mainly in the degree of apical expansion and approximately parallel sides of the tegmen), there is inadequate consistency to support multiple species at present. More careful study over this species’ range may conclude otherwise. Specimens from the Cochabamba region of Bolivia frequently exhibit anterior fragments of a lateral submarginal pronotal stria, but we have dissected these as well and find them to fit within this broad concept of *P.thiemei*. *Phelisterrufinotus* occurs broadly over much of the same range as this species, but we have generally had little difficulty separating them, on the basis of (in *P.rufinotus*) a partial lateral submarginal pronotal stria, and the spoon-shaped aedeagus.

##### Biology.

Label data provide limited clues into the habits of this species; a few specimens were collected in cow dung or in pitfalls baited with human dung. Numerous specimens were simply collected by flight interception traps.

##### Distribution.

This species is known from a fairly broad area from southeastern Bolivia and southeastern Brazil in the north through Uruguay and Paraguay south across central Argentina. **Records: ARGENTINA**: Buenos Aires, Cordoba, Corrientes, Entre Rios, Mendoza, San Luis; **BOLIVIA**: Cochabamba, Santa Cruz; **BRAZIL**: Mato Grosso, Minas Gerais, Rio Grande do Norte, Rio Grande do Sul, São Paulo; **PARAGUAY**: Caazapá, Misiones; **URUGUAY**: Canelones, Rocha; **VENEZUELA**: Aragua.

**Map 5.**
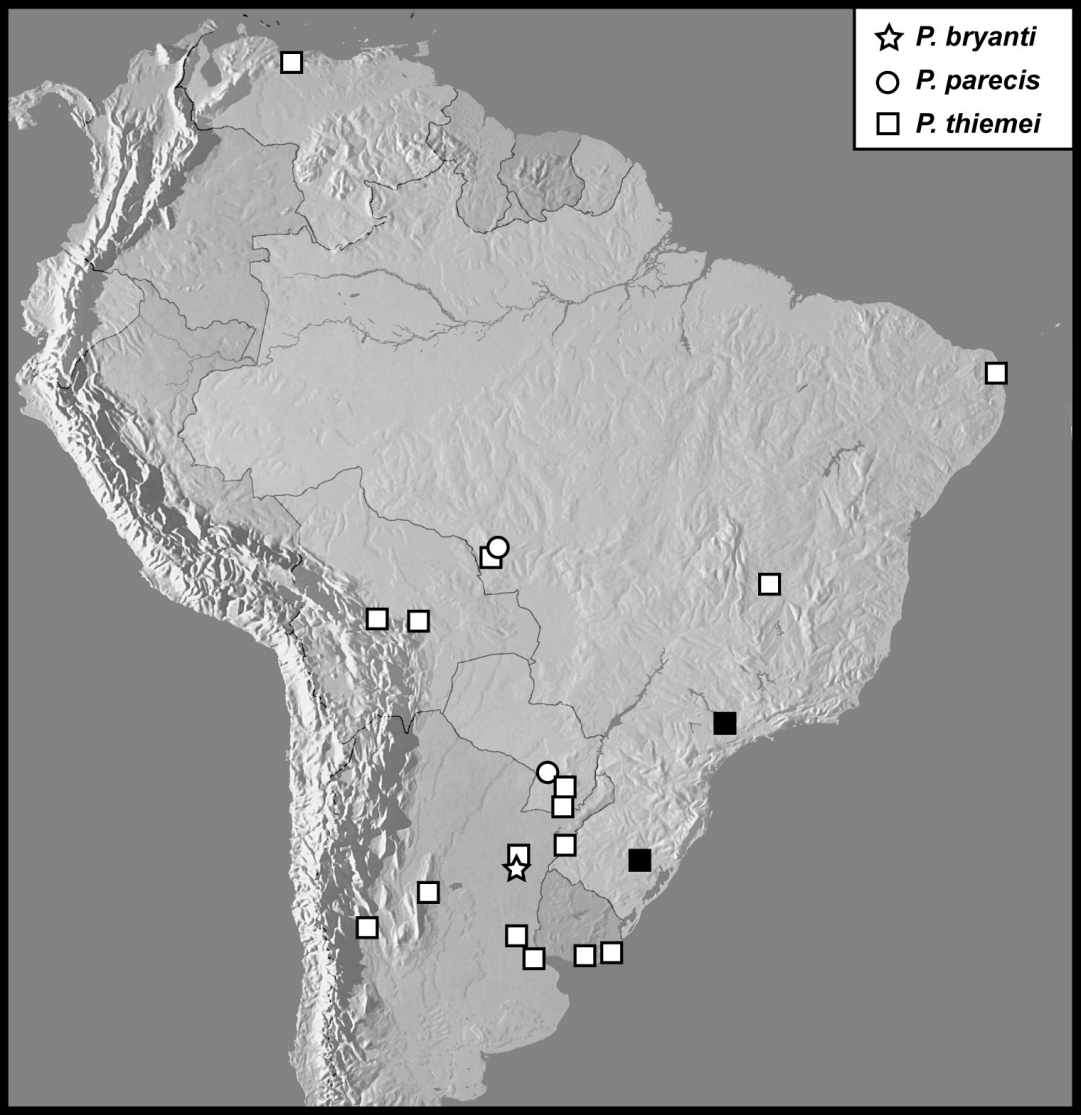
Collecting records for *Phelisterbryanti* (stars), *P.parecis* (circles), and *P.thiemei* (squares; filled squares represent state records only for *P.thiemei*).

#### Phelister parecis sp. nov.

Taxon classificationAnimaliaColeopteraHisteridae

http://zoobank.org/5F177312-1062-4453-940C-7035402D19F0

Figs 2 , 8 ; Map 5

##### Type material.

**Holotype male**: “BRASIL: Mato Grosso, Chapada dos Parecis, 14°15.85'S, 59°14.03'W, 25.xi-16.xii.2000” / “Caterino Tischechkin Exosternini Voucher EXO-00146”, deposited in DZUP. Paratypes (5): 2: Same data as type (CHND, FMNH); 1: PARAGUAY: Paraguari, Compañía Naranjo, 5 November 2000, C. Aguilar” / “Caterino Tischechkin Exosternini Voucher EXO-00147”; 2: PARAGUAY: Cordillera, Naranjo, 3.xi.2000, C. Aguilar” (CHND, MSCC).

##### Diagnostic description.

Length: 1.34–1.62 mm (avg. 1.48 mm); width: 1.14–1.30 mm (avg. 1.26 mm). Body elongate oval, dark rufescent to piceous, the elytra more distinctly rufescent; frons depressed along midline, lacking secondary punctures, with complete frontal stria; labrum weakly emarginate; both mandibles with strong inner marginal tooth; pronotum with fine but distinct ground punctation, with coarser punctures in the lateral thirds, as well as along the basal margin; prescutellar impression distinct; marginal pronotal stria complete around sides and front; lateral submarginal pronotal stria complete along sides, very close to marginal stria, curving inward at front, nearly merging with marginal stria behind eye, strongly crenulate; elytra with single, complete epipleural stria, outer subhumeral stria present in posterior one-half, inner absent, dorsal striae 1–5 complete, the 5^th^ hooked weakly at base, sutural stria present in apical two-thirds; propygidium with conspicuous, round secondary punctures separated by ca. their diameters, only slightly smaller and sparser in apical half; pygidium with very small, sparse secondary punctures throughout; prosternal lobe bluntly rounded, with complete marginal stria; prosternal keel with complete striae, sinuate, united anteriorly, with denser intervening punctures in the male; mesoventrite moderately produced, with complete marginal stria close to margin, continued at sides by long postmesocoxal stria that extends two-thirds of the distance to the posterior corner of the metepisternum; mesometaventral stria weakly arched onto base of mesoventrite, angulate at sides, lateral metaventral stria extending nearly to middle of front edge of metacoxa; 1^st^ abdominal ventrite with complete inner lateral stria and fragments of outer behind metacoxa; protibia with outer edge rounded, bearing four moderately strong teeth, with prominent spines, apex obliquely truncate; protarsal claws unmodified; meso- and metatibiae weakly expanded to apices, bearing marginal spines, principally in the apical half on the metatibia; basal piece ca. one-fourth aedeagus length; tegmen narrow, only weakly expanded to apex, not very dorsoventrally flattened, rather thick in apical half; median lobe over half tegmen length, proximal apodemes differentiated with thin basal portions long.

**Figure 8.**
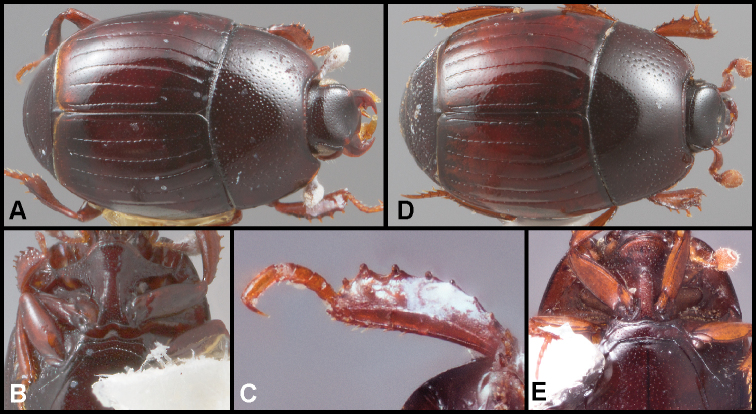
**A–C***Phelisterbryanti* sp. nov. **A** Dorsal habitus **B** Prosternum and mesoventrite of male **C** Bent protarsal claws **D–E***Phelisterparecis* sp. nov. **D** Dorsal habitus **E** Ventral view showing prosternal and meso- and metaventral striae.

##### Remarks.

This species is very closely related to the *P.rufinotus* complex, and we considered the possibility that it represented a variant of one of these. But it is consistently distinct, over several localities, in the complete lateral submarginal pronotal stria, the complete 5^th^ dorsal stria, and the complete frontal stria. Its aedeagus (from only one available male) is narrower than others in this complex, as well.

##### Biology.

Nothing is known of the biology of this species.

##### Distribution.

This species is known from only three locations, from Mato Grosso, Brazil to southern Paraguay.

#### Phelister bryanti sp. nov.

Taxon classificationAnimaliaColeopteraHisteridae

http://zoobank.org/3B92666E-C64C-4A82-98C0-510275B0D62A

Figs 2 , 8 ; Map 5

##### Type material.

**Holotype male**: “Rio San Javier, Santa Fe, Argentine. G.E. Bryant. San Joaquin 5.1.1912” / “G. Bryant Coll. 1919-147” / “Phelister subrotundus Say, H. Desbordes det. 1932” / “Caterino Tischechkin Exosternini Voucher EXO-00144”, deposited in NHMUK. 2 paratypes, same data as type (NHMUK).

##### Diagnostic description.

Length: 1.50–1.77 mm (avg. 1.62 mm); width: 1.22–1.38 mm (avg. 1.29 mm). Body broadly elongate oval, piceous, with conspicuous ground punctation, especially on pronotum; frons depressed along midline, lacking secondary punctation, with complete supraorbital stria; frontal stria obsolete between antennal bases; labrum moderately emarginate apically; both mandibles with strong inner marginal tooth; pronotal disk with few coarser secondary punctures at sides of disk and along basal margin; prescutellar impression present, but small; marginal pronotal stria complete along sides and front, crenulate anteriorly; submarginal pronotal striae absent; elytra with single, complete epipleural stria; outer subhumeral stria present in apical half only, inner absent, elytral striae 1–5 complete, sutural stria present in apical two-thirds or slightly more; propygidium with few sparse secondary punctures, mostly in basal half; pygidium with ground punctures only; prosternal lobe bluntly rounded, with complete marginal stria; prosternal keel with complete striae parallel over most of length, slightly divergent basad, connected basally by transverse stria, free anteriorly; mesoventrite moderately strongly produced, with complete marginal stria, continued at sides by postmesocoxal stria which diverges sinuately onto metaventrite; mesometaventral stria subangulate at middle, reaching midpoint of mesoventrite, curving posteriad at sides rather distant from mesocoxa, continued by lateral metaventral stria nearly to middle of metacoxa; 1^st^ abdominal ventrite with single, complete lateral stria; protibia with outer margin weakly rounded, widest near middle, with 5–6 weakly developed teeth bearing marginal spines, apex obliquely truncate; protarsal claws (of male only?) strongly bent at base, straight to apex; meso- and metatibiae elongate, thin, mesotibia with ca. five thin marginal spines, those of metatibia very fine and mostly near apex; basal piece ca. one-fourth total aedeagus length; tegmen moderately flattened dorsoventrally, lacking ventral process, sides widening to near apex, then abruptly narrowed to thin, elongate apices, apical emargination narrow; median lobe ca. one-third tegmen length, with proximal apodemes differentiated into thin and longer thick portions.

##### Remarks.

This species appears quite similar to *P.puncticollis*, but is distinct in lacking a sublateral pronotal stria, its impunctate metaventrite, and separate male prosternal striae. The aedeagus of *P.bryanti* is highly distinct, being abruptly narrowed apically, where that of *P.puncticollis* is narrow and evenly rounded to the apex.

##### Etymology.

This species is named for the collector of the entire type series, GE Bryant, a British coleopterist, best known for his work on Chrysomelidae.

##### Biology.

Nothing is known of the biology of this species.

##### Distribution.

This species is only known from the type locality in northeastern Argentina, and the types’ labels bear no ecological data.

#### Phelister vernus

Taxon classificationAnimaliaColeopteraHisteridae

(Say, 1825)

Figs 2 , 9 ; Map 6

Hister vernus Say, 1825: 40.
Phelister vernus ; Marseul, 1853: 478.
Phelister saunieri Marseul, 1861: 162; Bickhardt, 1916: 214.

##### Type material.

Neotype of *Histervernus* Say, hereby designated: [reddish round disk] / “H. vernus Say.” / “NEOTYPE Hister vernus Say Desg. Caterino and Tishechkin, 2011”, MCZC. This common, widespread, and somewhat variable Nearctic species needs to be represented by a physical type so as to precisely establish its identity.

Types of synonyms. **Lectotype** of *Phelistersaunieri* Marseul hereby designated: “11 Phelister saunieri M vernus Say Rochester Bouses Mai” / “6(11^a^) Phelist. Saunieri M Et Un. Rochester” / “TYPE” / “MUSEUM PARIS Coll. de Marseul 2842-90” / “LECTOTYPE Phelister saunieri Marseul, 1861 M.S. Caterino and A.K. Tishechkin des. 2010”, MNHN.

##### Diagnostic description.

Length: 1.73–2.05 mm (avg. 1.95 mm); width: 1.50–1.77 mm (avg. 1.62 mm). Body elongate oval, piceous, with very distinct ground punctation throughout, especially distinct on pronotum; frons depressed along midline, supraorbital stria complete, frontal stria present along eyes, but broadly interrupted across front; labrum shallowly emarginate at apex; mandibles both with strong inner marginal tooth; prescutellar impression distinct, posterior margin of pronotum also with crenulate marginal punctures; lateral one-fifth of pronotal disk with elongate, coarser punctures; marginal pronotal stria complete along sides and front, only weakly crenulate in front; submarginal stria absent; marginal epipleural stria complete; outer subhumeral stria present in posterior one-third, inner subhumeral absent; dorsal elytral striae 1–4 complete, 5^th^ present in apical half (sometimes also as basal puncture), sutural stria present in apical two-thirds; propygidium with small secondary punctures interspersed with ground punctures, separated by ca. twice their diameters; pygidium with ground punctures only; prosternal lobe narrowly rounded, subtruncate apically, marginal stria somewhat distant from margin, may be interrupted at middle; prosternal keel with striae converging from base, subparallel in apical half, similarly punctate in both sexes; mesoventral stria sinuate, following anterior margin, deeply impressed, continued by postmesocoxal stria, curving laterad behind coxa; mesometaventral stria somewhat quadrate, weakly arcuate across base of mesoventrite, angulate posteriad, with lateral mesoventral striae only weakly diverging to inner corners of metacoxae; 1^st^ abdominal ventrite with complete inner lateral stria, outer abbreviated at base, and diverging behind metacoxa; protibia with outer margin weakly rounded, and rather strongly dentate, with five marginal spines, apex truncate; meso- and metatibiae weakly expanded to apex, mesotibia with ca. five marginal spines, more prominent toward apex, metatibia with distinct spines mainly in apical half. Aedeagus with basal piece almost one-third aedeagal length, tegmen more or less parallel sided, rounded to apex, with narrow, closed apical emargination; in lateral view tegmen rather flattened, thickened and with ventral dentate process near its midpoint; median lobe short, ca. one-third tegmen length, with differentiated thick and thin proximal apodemes.

**Figure 9.**
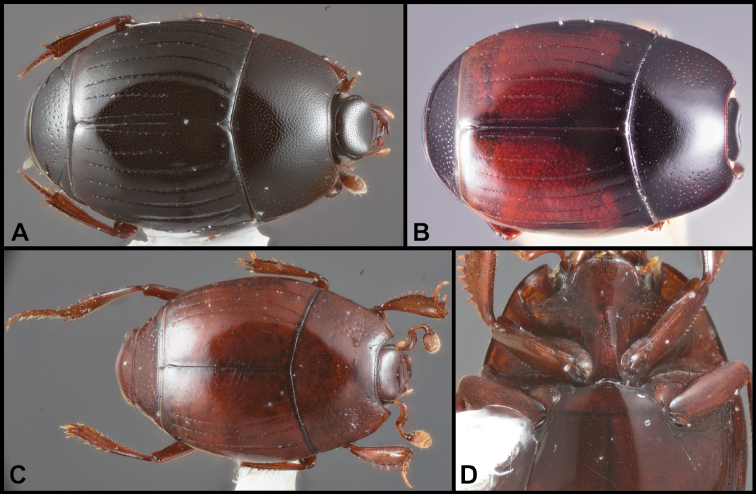
**A***Phelistervernus* (Say), dorsal habitus **B***Phelisterchilicola*, dorsal habitus **C–D***Phelisterbruchi* Bickhardt: **C** Dorsal habitus **D** Ventral view showing prosternal and meso- and metaventral striae.

##### Remarks.

Among US species of *Phelister*, *P.vernus* is easily distinguished by its broadly interrupted frontal stria, its lack of submarginal pronotal stria, and its conspicuous pronotal ground punctation. It also lacks any hint of reddish coloration, common in the broadly sympatric *P.subrotundus.*

##### Biology.

Label data associated with specimens indicate varied habits, having been collected from cow dung, mushrooms, ‘stable sweepings’, and even in the nest of a shrew.

##### Distribution.

There is a published record from Ontario (Davies 1991), but neither we nor Bousquet and Laplante (2006) have seen any specimens from Canada. Otherwise, the species is common and widely distributed across the eastern Nearctic. **Records: USA: Alabama**: Blount, Dallas, Madison, Mobile; **Arkansas**: Pulaski, Newton, Polk, Washington; **Connecticut**: New London; **District of Columbia**; **Florida**: Alachua, Columbia, Hendry, Putnam, Suwanee, Volusia; **Georgia**: Clarke, Chattooga, Dade, Harris, Peach; **Illinois**: Champaign, Cook, Dupage, Jackson, Lake, Lee, McClean, Will; **Indiana**: Tippecanoe, Vanderburgh; **Iowa**: Johnson; **Kansas**: Douglas, Jefferson, Leavenworth, Riley, Shawnee; **Kentucky**: Franklin, Jefferson; **Louisiana**: Calcasieu, East Baton Rouge, Grant, Jefferson, Madison, Orleans, Pointe Coupee, St. Charles, St. Tammany, West Feliciana; **Maryland**: Prince George’s, St. Mary’s; **Mississippi**: Hinds, Issaquena, Oktibbeha, Panola, Pontotoc; **Missouri**: Carter, Lawrence; **Nebraska**: Lancaster; **New Jersey**: Essex, Passaic; **New York**: Kings, Orange, Queens, Suffolk; **North Carolina**: Buncombe, Jackson, Rockingham; **Oklahoma**: Latimer; **Pennsylvania**: Delaware, Lancaster, Luzerne, Northampton, Philadelphia; **South Carolina**: Anderson, Charleston, Dorchester, Florence, Horry, Lexington, Pickens; **Tennessee**: Benton, Davidson, Knox, Sevier; **Texas**: Bexar, Brazos, Burleson, Cameron, Collin, Colorado, Dallas, Fort Bend, Gillespie, Guadalupe, Hidalgo, Jim Wells, Sabine, Travis, Uvalde, Wood; **Virginia**: Fairfax, Lee, Nelson; **West Virginia**: Hampshire, Jackson, Mason, Pocahontas; **Wisconsin**: Kenosha.

**Map 6.**
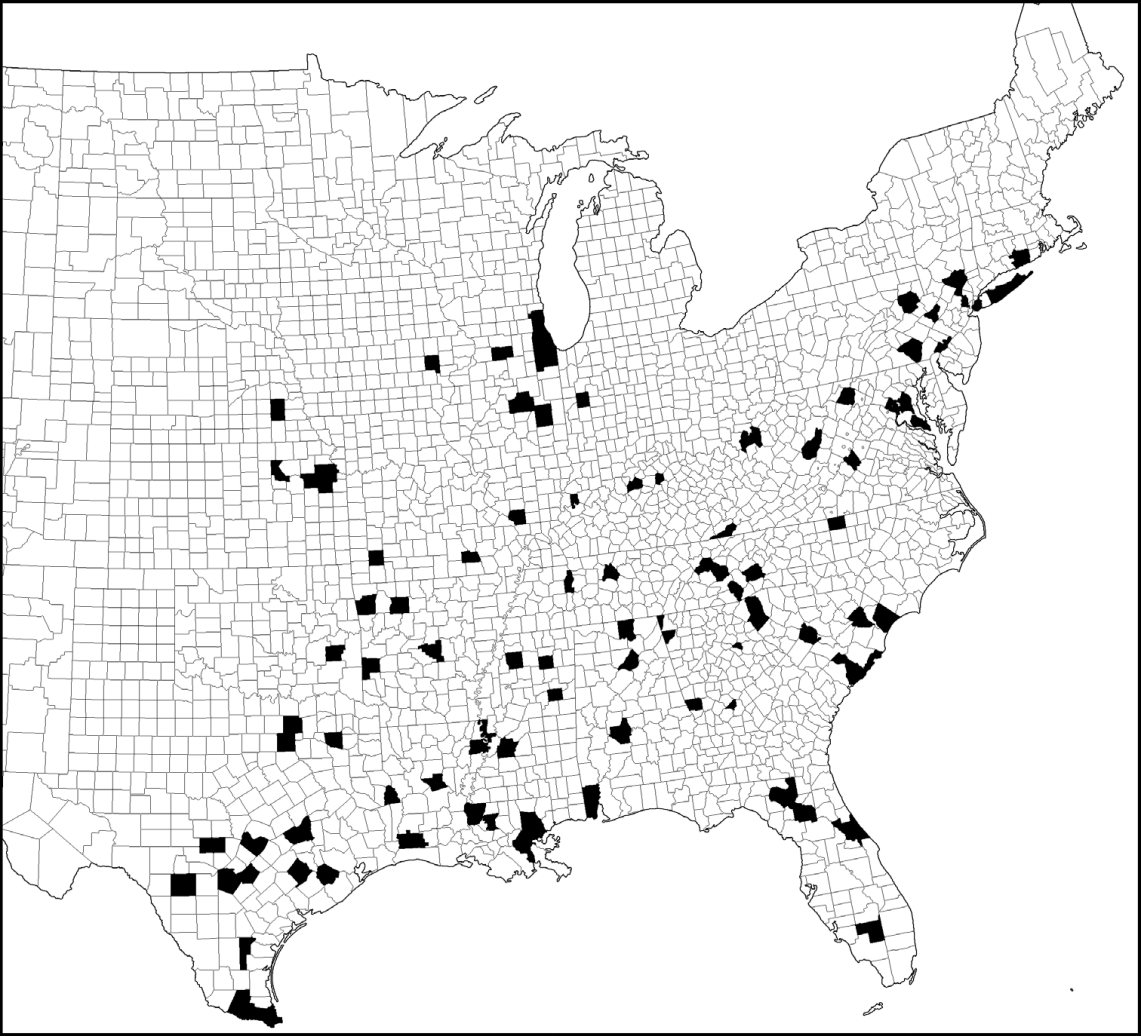
United States county map shading counties with records of *Phelistervernus*. No records have been confirmed for either Canada or Mexico.

#### Phelister chilicola

Taxon classificationAnimaliaColeopteraHisteridae

Marseul, 1870

Figs 2 , 9 ; Map 7

Phelister chilicola Marseul, 1870: 79.

##### Type material.

**Lectotype**, hereby designated: “Phelister chilicola, Chili, [?????] 68” / “Museum Paris, Coll. de Marseul 2842-90”/ “Type”/ “Lectotype Phelister chilicola Marseul, 1870, M.S. Caterino & A.K. Tishechkin des. 2010”, MNHN.

##### Diagnostic description.

Length: 1.73–2.17 mm (avg. 2.02 mm); width: 1.50–1.81 mm (avg. 1.66 mm). Body elongate oval, widest behind humeri, piceous with the apices of the elytra and the legs typically castaneous to rufescent, the ground punctation fine but distinct; frons depressed along midline, lacking secondary punctures; supraorbital stria complete, frontal stria fine, interrupted at middle for ca. width of labrum, inner ends weakly recurved dorsad; labrum weakly emarginate; mandibles both with distinct inner marginal tooth, that of left mandible slightly larger; pronotal disk with few coarser secondary punctures at sides and row of coarse punctures along posterior margin; prescutellar impression present but weak; marginal pronotal stria complete along sides and front, not distinctly crenulate anteriorly; submarginal pronotal stria absent; elytra with single, complete epipleural stria, outer subhumeral stria present in apical half, inner subhumeral stria absent; dorsal striae 1–4 complete, 4^th^ rarely abbreviated at base, 1 and 2 weaker apically, 5^th^ stria present in apical half, sutural stria present in apical two-thirds; propygidium with small, sparse secondary punctures, mainly in basal half; pygidium with ground punctation only; prosternal lobe evenly rounded, with complete marginal stria; prosternal lobe with two complete striae converging slightly toward front, the intervening punctures not sexually dimorphic in density; mesoventrite weakly projecting, with complete marginal stria, continued at sides by postmesocoxal stria which diverges to sides, ending freely before reaching middle of metepisternum; mesometaventral stria straight to angulate at middle, often reaching middle of mesoventrite, continued at sides by well-impressed lateral metaventral stria which reaches middle of metacoxa; 1^st^ abdominal ventrite with complete inner lateral stria, the outer generally abbreviated from both base and apex; protibia with outer margin distinctly rounded, widest near middle, with five weakly developed teeth bearing marginal spines; protarsal claws of both sexes strongly bent at base, then straight; meso- and metatibiae evenly widened to apices, with few weak marginal spines confined to apical halves; basal piece of aedeagus ca. one-fourth entire aedeagus length; tegmen dorsoventrally flattened, with weak ventral process ca. one-third from base, tegmen widening toward apex, sides rounded, apices bluntly rounded, with narrow, rather shallow apical emargination; median lobe a little over one-half tegmen length, proximal apodemes thin at bases, thicker over apical two-thirds.

##### Remarks.

This species is superficially similar to other red-marked species, such as *P.haemorrhous*, *P.rufinotus*, and *P.thiemei*. However, it is clearly and easily distinguished from any of these by its thin elytral striae, modified protarsal claws (in both sexes), and more diffuse reddish coloration of the elytra only. The aedeagus is also quite distinct from any of these, particularly in the obvious ventral process of the tegmen, and in the short proximal apodemes of the median lobe.

##### Biology.

None of the specimens we have seen include any biological data.

##### Distribution.

This species is known only from Central Chile, ranging from Santiago Province in the north to Valdivia in the south. **CHILE**: Cautín, Concepción, Nuble, Osorno, Santiago, Valdivia.

**Map 7.**
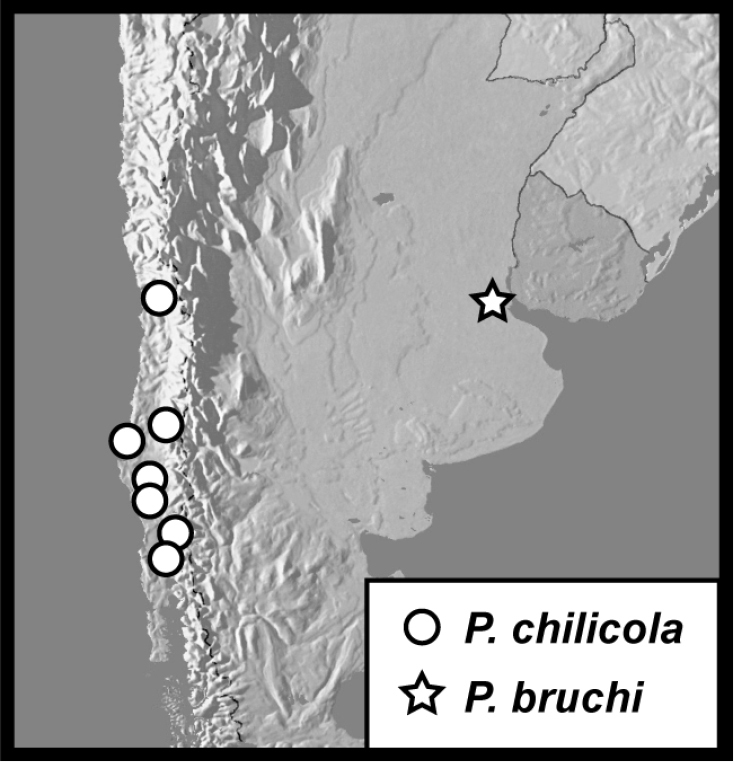
Collecting records in southern South America for *Phelisterchilicola* (circles) and *P.bruchi* (star).

#### Phelister bruchi

Taxon classificationAnimaliaColeopteraHisteridae

Bickhardt, 1920

Figs 2 , 9 ; Map 7

Phelister bruchi Bickhardt, 1920: 237.

##### Type material.

**Lectotype** of undetermined sex, hereby designated: “Rep. Argentina, Prov. Buenos Aires 17.X.1919” / “ex nido de Ctenomys” / “Phelister Bruchi Bickh. H. Bickhardt det. 1920.” / “LECTOTYPE Phelister bruchi Bickhardt, 1920 M.S. Caterino and A.K. Tishechkin des. 2010”, ZMHB. 8 paralectotypes designated (on 6 pins) with same data as lectotype, ZMHB. There are two other probable syntypes (unmarked and undesignated) in FMNH.

##### Diagnostic description.

Length: 2.01–2.05 mm (avg. 2.04 mm); width: 1.73–1.77 mm (avg. 1.76 mm). Body elongate oval, moderately depressed, rather pale rufescent; frons with fine ground punctation, weakly depressed at middle, frontal stria interrupted briefly at middle; labrum deeply emarginate, apical margin subcarinate; mandibles both strongly toothed along inner edge; pronotum with sides strongly convergent, only weakly curved, disk impunctate at middle, with sparse larger punctures at sides; prescutellar impression very small, fine; punctures along basal margin weak; marginal pronotal stria complete along sides and front; submarginal striae absent, but three gland openings conspicuous along lateral margins; elytra with single, complete epipleural stria, outer subhumeral stria very short and apical, inner subhumeral stria absent; dorsal elytral striae 1–3 complete (3^rd^ may be weakly abbreviated apically), 4^th^ present in basal third, and maybe as apical fragments, 5^th^ and sutural striae absent; propygidium with sparse small punctures separated by 2–3× their diameters, also with faint wavy microsculpture near base; pygidium with only very small and ground punctation; prosternal lobe rather elongate, with complete marginal stria; prosternal keel narrow, lacking striae; mesoventrite projecting, with marginal stria fine, merging with margin at middle, thus appearing interrupted; mesometaventral stria absent from mesoventrite; postmesocoxal stria present curving strongly laterad behind coxa; lateral metaventral stria present, extending from inner margin of mesocoxa ca. two-thirds the distance to outer corner of metacoxa; 1^st^ abdominal ventrite with only weak fragments of a lateral stria; all legs rather elongate and slender; protibia with lateral margin rounded, with 6–7 marginal spines, apex obliquely truncate; protarsi elongate, with almost straight protarsal claws; meso- and metatibiae narrow and elongate, with rather fine, elongate marginal spines, those of metatibia restricted to apical half; basal piece short, ca. one-sixth total length of aedeagus narrow, sides subparallel, apices bluntly rounded, with shallow apical emargination; median lobe long, ca. four-fifths tegmen length, proximal apodemes differentiated into thick and longer thin portions.

##### Biology.

This species has only been collected once to our knowledge, from burrows of *Ctenomys* Blainville (tuco-tucos). Its habitus, with long thin legs and weakly impressed striae, reflects its probable status as an obligate inquiline in these burrows.

##### Distribution.

This species is only known from the type locality, in Buenos Aires province, Argentina.

## Results and discussion

**Figure 10.**
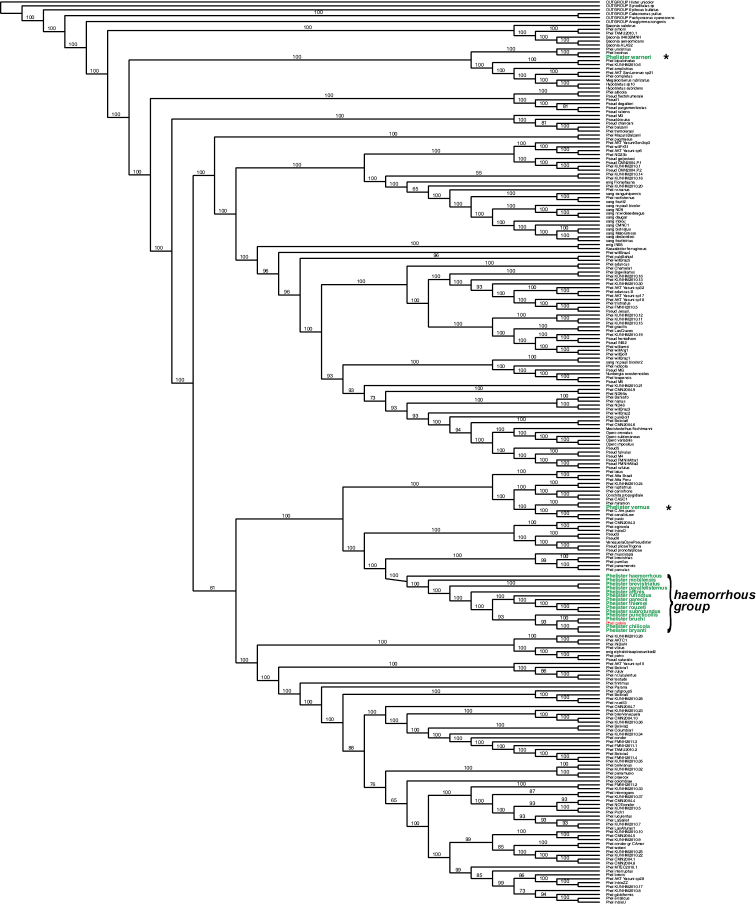
Majority rule consensus of 1000 trees of 10766 steps from parsimony search. Majority rule consensus indices are shown on branches. Taxa that are highlighted in green are members of the *Phelisterhaemorrhous* group, as delimited in this paper (including two not resolved to be part of the same clade, *P.vernus* and *P.warneri*). One species, *P.pulvis*, resolved among *P.haemorrhous* group species but excluded from the group is highlighted in red.

In our reduced analysis of *Phelister* (sensu lato) a single ‘best’ island of trees was found (saving the maximum 2500 trees) of 10777 steps. Continuing with an unconstrained search, beginning with these shortest starting trees, one shorter island of trees of 10766 steps was found. The majority rule consensus of these is presented in Figure 10. Fifteen of the included species are resolved in a single clade. However, there are two problems. The first is that two species that we have included in the *haemorrhous* group based on overall morphology do not fall in it in these analyses. Omission of one of these, *Phelistervernus*, corresponds to some of our own uncertainties with its assignment. It instead falls out with a Central American group of *Phelister* species that includes *P.pusio* Erichson, *P.canalis* Lewis, and *P.miramon* Marseul. The latter placement is somewhat intriguing, in that *P.miramon* was considered initially to be *P.vernus* (in Marseul’s writing on his own label for the type of *P.miramon*). However, we have studied type(s) of *P.miramon* (Caterino & Tishechkin, unpublished notes), and, despite some external similarities, it represents a distinct species with a very different type of aedeagus from anything in the *P.haemorrhous* group. This phylogenetic result may be driven more by superficial external characters than more significant internal ones. More surprising is the wide separation of our new species *P.warneri* from the *P.haemorrhous* group. This may also be driven by some unique external characters associated with its inquilinous habits, as its aedeagus shares several characteristics with other species in the *haemorrhous* group (especially *P.brevistriatus* and *P.sonorae*, which we suggest to be its closest relatives.)

The second problem is that *Phelisterpulvis* Marseul falls among members of the *P.haemorrhous* group, though we have not included it in this revision. It is difficult to see the basis for its inclusion, as its external similarity is minimal, lacking most of the characters that we list above as diagnostic for the *haemorrhous* group. This unlikely result is probably driven by the lack of male genitalic data available for *P.pulvis*, hindering its placement with species we do consider to be its more likely relatives.

Within the *haemorrhous* group, *P.haemorrhous* itself is resolved as sister to all other members of the group, with the North American *P.mobilensis* then sister to the remainder. *Phelisterbrevistriatus* and *P.parallelisternus* are united as sister groups, somewhat surprisingly to the exclusion of *P.affinis*. What we informally refer to as the ‘*rufinotus* complex’ is resolved as a clade. Remaining relationships show relatively little correspondence with obvious morphological characters, and demand more comprehensive analysis. Only four of these species are yet represented by any molecular data (*P.haemorrhous, P.subrotundus, P.rufinotus*, and *P.vernus*). More comprehensive data will be necessary to reveal more valuable insights into relationships.

## Supplementary Material

XML Treatment for Phelister haemorrhous

XML Treatment for Phelister affinis

XML Treatment for Phelister parallelisternus

XML Treatment for Phelister mobilensis

XML Treatment for Phelister brevistriatus

XML Treatment for Phelister sonorae

XML Treatment for Phelister warneri

XML Treatment for Phelister puncticollis

XML Treatment for Phelister subrotundus

XML Treatment for Phelister rouzeti

XML Treatment for Phelister rufinotus

XML Treatment for Phelister thiemei

XML Treatment for Phelister parecis

XML Treatment for Phelister bryanti

XML Treatment for Phelister vernus

XML Treatment for Phelister chilicola

XML Treatment for Phelister bruchi

## Supplementary materials

Supplementary material 1 Exosternini phylogenetic data Data type: (Morphological and molecular character state data) Explanation note: This dataset is a reduced (in numbers of taxa) from that published in Caterino and Tishechkin 2015. It contains 260 morphological characters followed by 2609 nucleotide characters, representing the 18S, 28S, and COI genes, scored for 231 taxa (including outgroups). This dataset is made available under the Open Database License (http://opendatacommons.org/licenses/odbl/1.0/). The Open Database License (ODbL) is a license agreement intended to allow users to freely share, modify, and use this Dataset while maintaining this same freedom for others, provided that the original source and author(s) are credited.Michael S. Caterino, Alexey K. Tishechkin
